# Single nucleotide polymorphisms to discriminate different classes of hybrid between wild Atlantic salmon and aquaculture escapees

**DOI:** 10.1111/eva.12407

**Published:** 2016-08-18

**Authors:** Victoria L. Pritchard, Jaakko Erkinaro, Matthew P. Kent, Eero Niemelä, Panu Orell, Sigbjørn Lien, Craig R. Primmer

**Affiliations:** ^1^ Department of BiologyUniversity of TurkuTurkuFinland; ^2^ Natural Resources Institute Finland (Luke)UtsjokiFinland; ^3^ Centre for Integrative Genetics (CIGENE)Department of Animal and Aquacultural SciencesNorwegian University of Life SciencesAasNorway

**Keywords:** allelotyping, aquaculture escapee, Atlantic salmon, introgressive hybridization, *Salmo salar*, SNP array

## Abstract

Many wild Atlantic salmon (*Salmo salar*) populations are threatened by introgressive hybridization from domesticated fish that have escaped from aquaculture facilities. A detailed understanding of the hybridization dynamics between wild salmon and aquaculture escapees requires discrimination of different hybrid classes; however, markers currently available to discriminate the two types of parental genome have limited power to do this. Using a high‐density Atlantic salmon single nucleotide polymorphism (SNP) array, in combination with pooled‐sample allelotyping and an F_st_ outlier approach, we identified 200 SNPs that differentiated an important Atlantic salmon stock from the escapees potentially hybridizing with it. By simulating multiple generations of wild–escapee hybridization, involving wild populations in two major phylogeographic lineages and a genetically diverse set of escapees, we showed that both the complete set of SNPs and smaller subsets could reliably assign individuals to different hybrid classes up to the third hybrid (F3) generation. This set of markers will be a useful tool for investigating the genetic interactions between native wild fish and aquaculture escapees in many Atlantic salmon populations.

## 1 Introduction

In common with many other salmonid fishes (e.g. Katz, Moyle, Quiñones, Israel, & Purdy, 2013; Metcalf et al., 2007; Rand, 2013), wild Atlantic salmon (*Salmo salar*) have declined over the past two centuries as a result of overfishing and habitat loss. The species has been extirpated from half of the major European river basins and a third of the major American river basins in which it historically occurred, and many of the remaining populations are considered to be under threat (ICES 2015; Parrish, Behnke, Gephard, McCormick, & Reeves, 1998). In contrast, since artificial cultivation began in Norway in the late 1960s, the captive Atlantic salmon population has exploded. In Europe over 1,000 times more salmon is currently produced by the aquaculture industry than is caught in the wild (ICES 2015). Most of these domestic Atlantic salmon are reared in open net‐pens in the marine environment, and escapes are frequent (Thorstad et al., 2008). In Norway, the world's biggest producer of farmed salmon, tens of thousands of aquaculture escapees, identified by body morphology and scale characteristics, are caught annually in the wild. Between 1989 and 2006, on average, 38% of the salmon catch of Norwegian coastal fisheries and 21% of the individuals sampled in spawning areas were aquaculture escapees (Thorstad et al., 2008).

Aquaculture salmon have undergone many generations of selection in the captive environment–both inadvertently, due to hatchery culture conditions, and deliberately. Some Norwegian aquaculture strains, for example, have been subject to artificial selection for multiple traits including speed of growth, weight and age at sexual maturity, and resistance to disease (Bicskei, Bron, Glover, & Taggart, 2014; Fleming, Agustsson, Finstad, Johnsson, & Björnsson, 2002; Gjøen, 1997). Correspondingly, farmed salmon have been shown to differ from their wild conspecifics in multiple traits including growth rate and age at maturation (Glover, Otterå, Olsen, & Slinde, 2009; Debes & Hutchings, 2014), migratory behaviour (Jonsson, Jonsson, & Hansen, 1991), heart rate and swimming endurance (Johnsson, Höjesjö, & Fleming, 2011), gene expression (Bicskei et al., 2014; Debes, Normandeau, Fraser, Bernatchez, & Hutchings, 2012) and response to stress (Solberg, Skaala, Nilsen, & Glover, 2013). These traits are expected to make aquaculture fish less well adapted to the natural environment, and multiple studies have demonstrated farmed salmon to have lower survival and reproductive fitness than native conspecifics in the wild (Fleming, Jonsson, Gross, & Lamberg, 1996; Jonsson & Jonsson, 2006; Naylor et al., 2005). Nevertheless, mature aquaculture escapees are frequently found in wild spawning areas (Erkinaro et al., 2010; Fiske, Lund, & Hansen, 2006) and can breed with wild fish (Clifford, McGinnity, & Ferguson, 1998). Introgressive hybridization – that is, the introduction of genetic material from the aquaculture escapees into the wild population via successive generations of interbreeding – poses a number of different threats to wild salmon, including the introduction of traits that are not locally adapted, a reduction in overall genetic diversity across populations, and the disruption of co‐adapted gene complexes that have become established within populations over evolutionary time (Edmands, 2006). The genetic contribution of aquaculture escapees to wild populations can be considerable: studies in Norway (Glover et al., 2012, 2013), Ireland (Clifford et al., 1998) and North America (Bourret, O'Reilly, Carr, Berg, & Bernatchez, 2011) have observed recent genetic changes in many wild Atlantic salmon populations that can be attributed to the influence of aquaculture escapees. However, other populations have maintained their genetic integrity despite large numbers of escaped fish being observed in their natal rivers (Glover et al., 2012, 2013). It is largely unknown what demographic or environmental factors influence the vulnerability of wild Atlantic salmon populations to genetic invasion by aquaculture escapees, although population density may play a role (Heino, Svåsand, Wennevik, & Glover, 2015).

Few sets of markers that can reliably discriminate the genomes of wild Atlantic salmon and aquaculture strains have been described (but see Karlsson, Moen, Lien, Glover, & Hindar, 2011), and this limits research into the dynamics of hybridization between escapees and wild fish. This relative paucity of markers is partly a function of the genetic characteristics of the aquaculture fish. Norwegian aquaculture lines, which are also farmed in a number of other countries, have mixed origins. They derive primarily from Norwegian populations in the Atlantic evolutionary lineage of *S. salar* with small contributions from populations in the North Barents/White Sea and Baltic evolutionary lineages (Bourret et al., 2013; Gjedrem, Gjøen, & Gjerde, 1991). Various different lines have been maintained separately since their initiation and are genetically very different from one another (Gjøen, 1997; Karlsson et al., 2011). These lines have both given rise to further sublines and been combined to form new lines (Karlsson, Diserud, Moen, & Hindar, 2014), and a salmon farm may use a varying mix of lines (Gjedrem et al., 1991; Gjøen, 1997; Thorstad et al., 2008). Further, the genetic composition and diversity of these aquaculture lines will have changed over time as a result of selection and drift and the addition of new material. Thus, there may be no consistent genetic signature whereby aquaculture escapees may be discriminated from wild fish.

**Figure 1:**
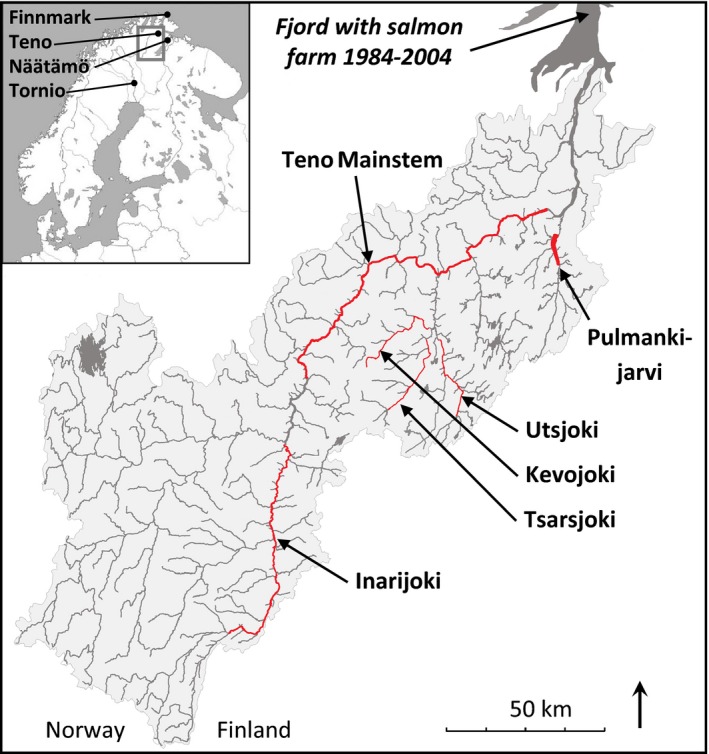
Sampling locations. Samples were collected by fisherman at multiple locations along the Teno mainstem, headwaters and tributaries

Using an array enabling the simultaneous genotyping of seven thousand (7K) single nucleotide polymorphism (SNP) markers in Atlantic salmon, Karlsson et al. (2011) identified a suite of 60 markers that discriminated major Norwegian aquaculture lines from wild Norwegian salmon. Collectively, these SNPs enabled identification of pure‐bred wild and aquaculture individuals and their F1 hybrids. However, they have not been shown to allow discrimination of different classes of later generation hybrids. This is imperative for a full understanding of the genetic interactions between wild and aquaculture salmon, but is a much more challenging analytical task (Vähä & Primmer, 2006). Here, we use an Atlantic salmon SNP array that includes 220,000 (220K) mapped SNPs, and combine it with a cost‐effective allelotyping approach, to identify a set of SNPs that can discriminate wild fish, a genetically diverse set of aquaculture escapees, and their first‐, second‐ and third‐generation hybrids. We focus on the Teno River of northern Finland and Norway, one of the world's largest and most diverse wild Atlantic salmon stocks (Vähä, Erkinaro, Niemelä, & Primmer, 2007; Erkinaro et al., 2010; Fig. 1). Reproductively mature aquaculture escapees have been caught throughout this river system since 1985 (Erkinaro et al., 2010). We further demonstrate that this SNP set performs well for hybrid class discrimination in additional populations, suggesting that it has general utility for examining wild–escapee hybridization over a wide geographical area.

## 2 Materials and Methods

### 2.1 Scale samples & DNA extraction

Archived Teno River Atlantic salmon scales, collected as part of a four‐decade monitoring program (Niemelä, Erkinaro, Julkunen, & Hassinen, 2005), were the primary source of genetic material for this study. All scales had been stored dry in envelopes prior to DNA extraction. As samples of aquaculture salmon, we used 240 aquaculture escapees captured in the Teno River between 1987 and 2010 (Erkinaro et al., 2010; hereafter referred to as ‘Teno Escapees’), and 228 escapees captured in the adjacent coastal waters of Finnmark, Norway, in 2008 and 2009 (hereafter referred to as ‘Finnmark Escapees’). All fish had been identified as escapees on the basis of morphological features and scale growth ring patterns consistent with previous captive rearing (Fiske, Lund, & Hansen, 2005). We expect these escapees to represent multiple different Norwegian strains of aquaculture salmon that are utilized in regional fish farms. As samples of wild Teno salmon unaffected by introgression from aquaculture escapees, we used individuals caught in the river between 1982 and 1987. Although a salmon farm was established near the mouth of the Teno in 1984, regional levels of salmon aquaculture were relatively low in the early 1980s, and the hybrid offspring of early escapes from this farm are not expected to return to the river before 1989 (Erkinaro et al., 2010). We chose not to use pre‐1982 samples due to the decrease in SNP genotyping quality with sample age when using similar SNP arrays (Johnston et al., 2013). Individuals were collected from the Teno mainstem (*n* = 120, hereafter referred to as ‘Old Teno Mainstem’), the Teno headwaters (Inarijoki, *n* = 114) and four tributaries: Kevojoki (*n* = 114), Pulmankijärvi (*n* = 114), Tsarsjoki (*n* = 114) and Utsjoki (*n* = 120) (Fig. 1). The salmon spawning in Kevojoki, Pulmankijärvi, Tsarsjoki and Utsjoki are known to be temporally stable, genetically distinct, populations (Vähä, Erkinaro, Niemelä, & Primmer, 2008; Vähä et al., 2007). The Teno mainstem has recently been shown to contain two overlapping subpopulations with low genetic divergence between them (Aykanat et al., 2015; Johnston et al., 2014); however, these were not separated for our study as the focus was on wild–aquaculture hybrid detection. The Inarijoki (headwater) population is genetically similar to the population in the upper Teno mainstem (Vähä et al., 2007). For the Old Teno Mainstem sample, we selected equal numbers of multi‐seawinter (MSW, 3 years at sea) and one‐seawinter (1SW, 1 year at sea) individuals (Johnston et al., 2014; Barson et al., 2015; sea age determined from scale growth ring patterns) from throughout the river. To obtain sufficient samples, we used scales from individuals that were caught from the last week of July onwards (6–8 weeks prior to spawning, Vähä et al., 2007) – thus, although the majority are expected to belong to the Teno mainstem populations, some may have been *en route* to other spawning locations. There are fewer multi‐seawinter fish spawning in Inarijoki and Teno tributaries (Vähä et al., 2007), and we only sampled 1SW fish in these locations. We selected an equal number of males and females within each location or seawinter class.

We extracted DNA from 2 to 4 scales per individual using a QIAmp DNA mini kit (Qiagen), following the manufacturer's protocol and with an initial Proteinase K digestion step. Older scale samples are known to yield more degraded DNA (Johnston et al., 2013) and therefore filter tips were used with pre‐2000 scales to minimize contamination risks. We assessed quality and concentration of all DNA extractions using a Nanodrop ND‐1000 spectrophotometer (Thermo Fisher Scientific Inc.). The Nanodrop method is known to overestimate concentrations when DNA is partly degraded (Simbolo et al., 2013); therefore, we also measured DNA concentration in a subset of samples (*n* = 14) via fluorometric quantitation using a Qubit 2.0 Fluorometer (Thermo Fisher Scientific Inc.).

Pilot studies showed that efficacy of Affymetrix SNP genotyping for older scales declined with the DNA concentration in the initial extraction: therefore, only extracts with >150 ng DNA/μl (as quantified by Nanodrop) were used further. Old Teno Mainstem and Teno Escapees were genotyped individually. Individuals from Teno headwater and tributaries, and Finnmark Escapees, were pooled by population for allelotyping, which is a cost‐effective approach for estimation of samplewide allele frequencies using SNP chips (Johnston et al., 2013; Ozerov et al., 2013; Sham, Bader, Craig, O'Donovan, & Owen, 2002). The following numbers of individuals were included in sample pools: Inarijoki 110, Kevojoki 102, Pulmankijärvi 83, Tsarsjoki 107, Utsjoki 108, Finnmark Escapees 227. Information on DNA concentration, estimated using Nanodrop, was used to equalize the amount of DNA contributed by each individual to the pool. To account for pipetting and allelotyping variability, six replicate pools were created (technical replicates) and two aliquots (subsamples) of each of these six pools were provided for analysis. For extracts from scales from the 1980s, there was an approximately linear relationship between DNA concentration estimated by Nanodrop and that estimated by Qubit, with Nanodrop estimates around five times higher than Qubit estimates. Therefore, for genotyping on the Affymetrix array we provided Old Teno Mainstem and Teno Escapee samples, and Teno pools, at a Nanodrop‐estimated concentration of 70 ng/μl. The Finnmark Escapee pool, containing DNA extracted from recently collected scales, was provided close to the Affymetrix recommended concentration (10 ng/μl), at a Nanodrop‐estimated concentration of 15 ng/μl.

For analysis and quality control purposes, we used data from three additional sets of scale samples: 530 Atlantic salmon individuals collected from the Teno mainstem between 2001 and 2003 as described in Johnston et al., 2013 & 2014 (hereafter referred to as ‘New Teno Mainstem’); 240 individuals collected between 2006 and 2008 from the Näätämö River of Finland and Norway, which is adjacent to the Teno; and 120 individuals collected between 2005 and 2008 from the Tornio River of Finland and Sweden, which flows into the Baltic Sea. New Teno Mainstem and Tornio fish were individually genotyped on the 220K array as part of the Barson et al. (2015) study. New Teno Mainstem individuals were also combined into four separate pools and allelotyped. Pooling of these samples was as described in Johnston et al., 2013: three pipetting replicates were generated per pool and three subsamples allelotyped per replicate. Näätämö samples were allelotyped: individuals were combined into four different pools each containing 60 individuals; four pipetting replicates were performed per pool, and each replicate allelotyped once.

### 2.2 Genotyping and allelotyping

A custom 220K Affymetrix Axiom array was used to allelotype or genotype samples on a GeneTitan genotyping platform, according to manufacturer's instructions (Affymetrix, USA). The SNPs on this array were a subset of those included on the 930K XHD *Ssal* array developed by T. Moen and colleagues (unpublished data), and had been chosen for maximum informativeness on the basis of their SNPolisher performance (SNPolisher, V1.4, Affymetrix), minor allele frequency (MAF) in aquaculture samples and physical distribution. All of these SNPs have a known location on the NCBI RefSeq Atlantic salmon genome (Lien et al., 2016, available: http://www.ncbi.nlm.nih.gov/genome/annotation_euk/Salmo_salar/100/). To ensure correct identification of genotype clusters, we applied the Affymetrix Best Practices Protocol for SNP calling simultaneously to a large data set that included the Old Teno Mainstem and Teno Escapee samples and all individuals genotyped for Barson et al. (2015). For allelotyping, pooled samples were subjected to the standard genotyping methodology, but normalized and summarized Allele A and Allele B probe intensities were returned instead of genotype calls.

### 2.3 Quality control of individually genotyped samples

One hundred and one of 112 Old Teno Mainstem samples, 199 of 239 Teno Escapee samples, 526 of 530 New Teno Mainstem samples and 117 of 120 Tornio samples passed quality controls on the Affymetrix array. Subsequent quality control steps were performed using plink v.1.90 (Chang et al., 2015; Purcell et al., 2007). First, we removed 1,112 SNPs not mapped to an assembled *S. salar* chromosome, 35 SNPs known to have off‐target variants, and 1,208 SNPs deviating from Hardy–Weinberg equilibrium at p < .0001 in the combined Old Teno Mainstem and New Teno Mainstem samples (indicative of technical genotype calling problems). Subsequently, we excluded 18,348 SNPs with >10% missing data or a MAF <10% in the combined Old Teno Mainstem and Teno Escapee data set. Finally, we excluded 17 individuals with >10% missing data. Following these quality control steps, 199,297 SNPs, genotyped in 94 Old Teno Mainstem, 192 Teno Escapee, 525 New Teno Mainstem and 115 Tornio individuals, were retained for analysis.

We examined genotyping repeatability in the Old Teno Mainstem samples by comparing genotype calls for four individuals that had been genotyped twice, using the *merge* function in plink; this was compared to the repeatability of five repeatedly genotyped New Teno Mainstem samples.

As an initial exploration of the genetic variation in the combined Old Teno Mainstem, New Teno Mainstem and Teno Escapee data set, we removed SNPs with >2% missing data, performed a linkage disequilibrium pruning step in plink (window size = 100, shift = 10, VIF = 2) and then used the *genome* function to calculate pairwise identity‐by‐state between all individuals, based on the remaining 48,375 SNPs. The presence of genotypic clusters was investigated by performing a two‐dimensional multidimensional scaling analysis (MDS) on the genomic identity‐by‐state (IBS) matrix in plink and visualizing the output using ggplot2 in R 3.1.2 (Wickham, 2009; R Core Team 2015, Fig. 2).

**Figure 2:**
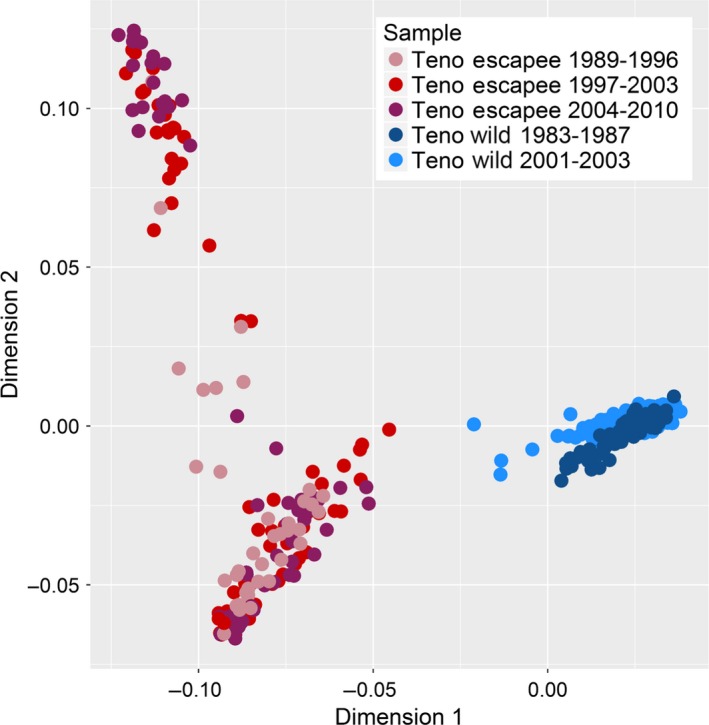
Multidimensional scaling analysis plot visualizing genomewide identity‐by‐state amongst Old Teno Mainstem, New Teno Mainstem and Teno Escapee samples. Each point represents an individually genotyped fish. Teno Escapee samples are colour‐coded by collection period

### 2.4 Estimation of allele frequency from pooled individuals

We estimated allele frequency at each SNP in each pool by calculating the relative intensity of the B‐allele probe signal (=B‐allele intensity/(A‐allele intensity + B‐allele intensity)), taking the median over all replicated pools, and applying a polynomial‐based probe‐specific (PPC) correction to account for differential hybridization efficiency of the two probes (Anantharaman & Chew, 2009; Brohede, Dunne, McKay, & Hannan, 2005). To obtain the PPC correction coefficients for each SNP, a second‐order polynomial describing the relationship between relative B probe intensity and genotype call (AA, AB or BB) was fit over the 525 individually genotyped New Teno Mainstem samples, plus 86 samples from the Teno mainstem genotyped for a different study, using a custom script in R. The polynomial with these coefficients was then used to correct the estimated allele frequency for that SNP in all allelotyped pools. SNPs with a PPC‐corrected allele frequency >1 or <0 were considered monomorphic, and allele frequencies were adjusted accordingly. The accuracy of the PPC‐corrected allele frequency estimates for the four New Teno Mainstem pools was investigated by regressing them against the true allele frequencies obtained from individual genotyping. Linear regression was performed using the *lm* function in R, specifying the model as (PPC‐corrected frequency) ~ 0 +  (True frequency) and using default parameters.

### 2.5 Selection of SNPs for hybrid class discrimination

To select SNPs potentially informative for hybrid class discrimination, we focused on regions of the genome that are unusually divergent between aquaculture escapees and wild Teno fish that were collected prior to aquaculture influence. To identify these regions, we used two different genome scan approaches which take population allele counts as input and use F statistics to identify outlying loci: Fdist2 (Beaumont & Nichols, 1996, available: http://www.maths.bris.ac.uk/~mamab/software/) and Bayescan (Foll & Gaggiotti, 2008). The source code for Fdist2 was slightly modified to allow a larger number of markers and compiled under Linux. Genomes scans were applied to the entire data set of 199,297 SNPs. Although our wild and aquaculture populations are expected to violate some of the model assumptions underlying these approaches (e.g. island migration model; independent divergence from a common ancestor), as our primary aim was to identify loci that could be used to discriminate between them rather than make inferences about differential selection we considered their use justified.

We converted allele frequency estimates from pooled samples into allele counts assuming 200 allele copies per locus per pool. For Fdist2 and Bayescan analyses, the ‘All Escapee’ sample was the combined allele counts of the individually genotyped Teno Escapees and the allelotyped Finnmark Escapee pool. Initially, this sample was compared to the combined ‘All Wild’ sample (allele counts from individually genotyped Old Teno Mainstem fish plus allele counts from pooled Inarijoki, Kevojoki, Tsarsjoki, Utsjoki and Pulmankijärvi). Subsequently, we compared the All Escapee sample each of the six Teno subpopulations separately. We ran Bayescan using default parameters. To apply Fdist2, we used the package function *datacal* to calculate observed heterozygosity and F_st_ for each locus. We then simulated the expected null distribution of heterozygosity and F_st_, assuming two demes/populations of 100 individuals, over 10,000,000 loci, using the function *fdist2*. In order to identify the model input value for *Expected F*_*st*_ that would generate a mean simulated F_st_ approximating the mean observed F_st_ between each escapee–wild comparison, we made pilot runs of 100,000 simulations iteratively changing *Expected F*_*st*_ until we obtained the desired values. Input values for *Expected F*_*st*_ were as follows: All Wild/All Escapee: 0.143; Inarjoki/All Escapee: 0.172; Kevojoki/All Escapee: 0.200; Pulmankijärvi/All Escapee: 0.207; Old Teno Mainstem/All Escapee: 0.115; Tsarsjoki/All Escapee: 0.361; Utsjoki/All Escapee: 0.228. To obtain the distribution of F_st_ values simulated by *fdist2* (within each H_e_ bin of 0.04), calculate empirical probabilities of our observed F_st_ values on the basis of these distributions, and convert these into *q* values, we used the R functions in *getPvalues.R*, written by Lotterhos and Whitlock (2014) and available in the Dryad repository (doi: 10.5061/dryad.v8d05).

All outlying loci identified by Bayescan or Fdist2 in all comparisons were examined for intrachromosome linkage disequilibrium using the *r* ^2^ function in plink, applied to the combined Old Teno Mainstem and New Teno Mainstem data sets. From these results, we identified clusters of physically adjacent linked SNPs: from observation of the data, a SNP was arbitrarily considered to be within a linked cluster when its pairwise *r* ^2^ with any other SNP within the cluster was >0.2.

We initially selected a set of 200 SNPs for use in hybrid discrimination, as pilot studies demonstrated no substantial increase in assignment efficacy using additional markers (data not shown), and because roughly this number of SNPs can be conveniently analysed on two 96‐well plates. We first chose SNPs located in linked clusters that were identified as outliers by both Bayescan and Fdist2 in the All Wild/All Escapee comparison. We chose one SNP per cluster, selecting the one that had the strongest outlying pattern over all the population comparisons. We supplemented these with SNPs in different linkage clusters that were identified as outliers by either Bayescan or Fdist2 in at least four comparisons between different Teno subpopulations and All Escapees.

### 2.6 Population genetic characteristics

For each population, mean expected heterozygosity was estimated from allele frequencies of the 199,297 SNPs using R. Pairwise unbiased F_st_ values between all populations (weighted by heterozygosity, Cockerham & Weir, 1993; Weir & Cockerham, 1984) were calculated from estimated allele counts using *datacal*. To examine whether use of pooled samples biased our estimation of F_st_ we calculated pairwise F_st_ between the All Escapee sample and each of the four New Teno Mainstem pools, first using allele frequencies obtained from individual genotyping and then using allele frequencies estimated using allelotyping. We also repeated all pairwise F_st_ comparisons using the subsets of 200, 160, 120, 80 and 40 loci selected for hybrid discrimination.

### 2.7 Simulation of hybrid populations

To test whether the 200 SNPs could in combination be used to discriminate hybrid classes, we used the Python package simuPOP (Peng & Kimmel, 2005) to simulate populations with allele frequencies at these SNPs approximating those observed in our genotyped and/or allelotyped samples (All Escapee, Old Teno Mainstem, Inarijoki, Kevojoki, Pulmankijärvi, Tsarsjoki & Utsjoki, plus the combined All Wild sample; see further details below), and ran them through three generations of hybridization. SimuPOP enables the user to simulate physical linkage between markers on the same chromosome by providing linkage distances between markers. Based on total physical length of the 29 assembled *S. salar* chromosomes (≈2200 million bp) and mean total linkage map distance estimated by Gonen et al., 2014 (2190 cM), we used the approximation 1 million bp = 1 cM.

Several sources of bias could lead us to overestimate the efficacy of our 200 SNPs to discriminate the genomes of wild fish and aquaculture escapees. First, we observed that using allele frequencies obtained via allelotyping caused F_st_ between the All Escapee and New Teno Mainstem pools to be overestimated by up to 7% (Table S1, see below). Second, the estimate of pairwise F_st_ from population samples is in general expected to be higher than the true population‐level value due to sampling variance (Anderson, Waples, & Kalinowski, 2008). In order to minimize these biases, we deliberately adjusted the allele frequencies in our simulated wild and escapee populations to be closer to one another. Initial B‐allele frequencies for the simulated populations were those estimated from genotyping/allelotyping the real population samples. First, based on a comparison of actual allele frequencies in the New Teno Mainstem pools to those estimated by allelotyping (Fig. S1), we adjusted allele frequencies of 0 or 1 to 0.025 or 0.975, respectively. For each locus in each population, we then generated a new B‐allele frequency by making random draws from the beta‐distribution with parameters (f*200, (1‐*f*)*200), where *f* is the B‐allele frequency following the previous adjustment step. Draws were performed using the R function *rbeta()*. For simulated Inarijoki, Kevojoki, Tsarsjoki, Pulmankijarvi, Utsjoki and All Wild populations, we chose the first value that adjusted the B‐allele frequency closer to the All Escapee value; for the simulated All Escapee population we chose the first value closer to the All Wild value. As Old Teno Mainstem allele frequencies were estimated from individual genotyping only, we did not adjust these values for the simulated Old Teno Mainstem population.

To create Generation 0 in simuPOP, we used the adjusted allele frequencies to initialize an escapee and a wild population each containing 500 individuals of each sex. We estimated pairwise F_st_ between the simulated Generation 0 populations by converting the adjusted allele frequencies into allele counts assuming 1,000 alleles per population and using these as input into the Fdist2 function *datacal*. We then simulated three generations of hybridization in simuPOP as follows: 100 individuals migrated from the escapee population into the wild population each generation; subsequently, 500 offspring of each sex were generated by random mating within each population, with parents sampled with replacement and each pair producing a single offspring. Individual IDs, parental IDs and genotypes for all individuals in all Generations (0 to 3) were output to a single file in (PED) format. We used a custom R script to calculate hybrid class for each individual by tracing its lineage from Generation 0. To facilitate comparison of hybrid class assignments amongst simulations with different ancestral wild populations, assignments were performed on standardized subsets of 400 individuals for each simulation. These subsets were generated by harvesting the same number of individuals of each hybrid class for each simulation. The number of individuals to be harvested was determined from the observed frequency of that hybrid class in the relevant generation over all simulations. For example, in Generation 2 overall frequencies of Pure Wild and Wild Backcross approximated 0.505 and 0.215, respectively; therefore, the test subset of 400 Generation 2 individuals for each wild ancestral population included 202 Pure Wild and 86 Wild Backcross individuals. Three possible Generation 3 hybrid classes (Escapee Backcross X Escapee Backcross, Escapee Backcross X F2, and F2 X F2) were excluded due to the small proportional representation of these classes. We also generated an independent set of Generation 0 individuals for each simulation (50 wild, 50 escapee) using the same initial allele frequencies. These individuals were created as wild and escapee reference samples and did not contribute to the hybridizing populations.

### 2.8 Hybrid class discrimination in simulated populations

We first used a Bayesian approach implemented in the program NewHybrids (Anderson & Thompson, 2002) to assign simulated individuals to user‐defined pure or hybrid classes (six possible classes for Generation 2; 21 possible classes for Generation 3, of which 18 were present in the data set and 14 could potentially be discriminated by NewHybrids). We provided the 100 reference individuals described above as reference samples: these individuals were not considered to be part of the hybrid test population. We additionally ran the Generation 2 analysis without a reference sample. We used the command‐line version of NewHybrids, compiled under Linux, and ran the analysis twice for each data set using different random seeds, with a burn‐in of 10,000 followed by 50,000 sweeps and default values for all other parameters. Number of sweeps was determined a priori by performing pilot runs in the graphical version of NewHybrids, with one of the Generation 3 data sets, using the same default parameters, and visually following the progress of the analysis. An individual was considered to be correctly classified when it was assigned to its own class with probability >0.5.

To examine the efficacy of hybrid class discrimination using fewer SNPs, we repeated the NewHybrid analyses with subsets of the initial 200 loci. Loci were ranked by allele frequency difference between the All Wild and All Escapee samples, and those with the smallest difference removed first. Results from analyses with *n* = 160, 120, 80 and 40 SNPs were compared to those from the full set of 200. We quantified the ability of different numbers of SNPS to correctly assign individuals to different hybrid classes, following Vähä and Primmer (2006), by defining the following measures. *Efficiency* is the proportion of individuals in a certain hybrid class that were actually assigned to that class by NewHybrids, for example (Total number of simulated F1 assigned to the F1 class)/(Total number of simulated F1). *Accuracy* is the proportion of individuals assigned to a class by NewHybrids that actually belong to that class, for example (Total number of simulated F1 assigned to the F1 class)/(Total number of all individuals assigned to the F1 class). *Overall performance* is the mean of *Efficiency* multiplied by *Accuracy* for each hybrid class over all populations.

As a supporting analysis, we also estimated the proportion of wild and escapee ancestry of each simulated individual using the command‐line version of the program Structure (Pritchard, Stephens, & Donnelly, 2000). Again, we provided reference samples of 50 wild fish and 50 escapees and defined them using ‘POPDATA’, ‘POPFLAG’ and ‘USEPOPINFO’ with MIGRPRIOR = 0.0001. We used *k* = 2, a burn‐in of 20,000 followed by 200,000 MCMC steps, recorded 95% confidence intervals of estimated ancestry, and retained default values for all other parameters.

From Structure results, five classes (pure wild, pure escapee, F1 or F2 hybrids, and backcrosses in each direction) in Generation 2, and nine classes in Generation 3, could be discriminated on the basis of expected admixture proportions. We assigned individuals to classes by examining their proportion of ancestry from the wild cluster. Individuals were considered to be assigned to a class when the 95% confidence intervals of their estimated wild ancestry did not overlap the expected mean ancestry of adjacent classes (e.g. an F1/F2 individual has an upper CL of wild ancestry <0.75 and a lower CL of wild ancestry >0.25; pure wild or pure escapee individuals had the 95% CL of their wild ancestry overlapping 1.0 and 0.0, respectively). We examined the assignment performance of different sets of SNPs as described above.

### 2.9 Hybrid discrimination in additional wild populations

As the discriminatory SNPs were selected based on allele frequency differences between the same populations that were subsequently used to test them, our results may overestimate the efficacy of SNPs to discriminate wild–escapee hybrids in other populations (‘high grading bias’, Anderson, 2010; Waples, 2010). We therefore tested the ability of these 200 SNPs to discriminate different classes of simulated hybrids using two additional wild populations. The Näätämö Atlantic salmon population belongs to same evolutionary lineage (North Barents/White Sea) as the Teno River, while the Tornio population is within a genetically divergent lineage (Baltic, Bourret et al., 2013, Fig. S9). For Näätämö, B‐allele frequency at the 200 SNPs was estimated by taking the median over all 16 allelotyped pools and applying the PPC correction; for Tornio, it was estimated from all 115 individually genotyped fish. Allele frequencies for Näätämö and Tornio were not further adjusted. Although we did not have an independent escapee sample, we made our analysis more conservative by estimating Generation 0 allele frequencies for our simulated escapee population from a randomly chosen subset of 90 Teno Escapees rather than the entire All Escapee sample. The remaining 99 Teno Escapee were used to estimate allele frequencies for the escapee reference sample. As before, three generations of hybridization were simulated using simuPOP, and NewHybrids used to assign simulated Generation 2 and Generation 3 individuals to different hybrid classes as described above.

## 3 Results

### 3.1 Initial exploration

Overall, Old Teno Mainstem samples, collected in the 1980s, failed Affymetrix quality controls more frequently and exhibited more missing genotypes than New Teno Mainstem samples, collected in the 2000s. Mean genotype repeatability over the four replicate Old Teno Mainstem samples was 98.0%, as compared to 99.3% over the five replicate New Teno Mainstem samples.

Preliminary exploration of genomewide patterns of identity‐by‐state revealed clear genetic differentiation between the individually genotyped Teno Mainstem and Teno Escapee samples (Fig. 2). Further, the escapee sample comprised two, largely discrete, genetic clusters. These clusters were not related to the time period in which the escapees were collected, and overall, we observed no clear temporal patterns in the genomic composition of the escapee or Teno samples. Three individuals classified as ‘aquaculture escapees’ clustered with the wild Teno individuals and were considered misidentified and removed (Fig. S9). No individuals classified as wild Teno fish clustered with the aquaculture escapees; however, several New Teno Mainstem individuals located between the Teno and Escapee clusters were considered potential wild–escapee hybrids (Fig. 2).

### 3.2 Accuracy of allele frequency estimation by allelotyping

For the 199,297 SNPs that remained after filtering, population allele frequencies estimated from the four New Teno Mainstem pools with the PPC correction were closely linearly related to the true frequencies estimated by individually genotyping (Fig. S1; each pool: *r* ^2^ = .996, residual SE = 0.037–0.039).

### 3.3 Population genetic parameters

Mean H_e_ within the sampled populations, calculated over all 199,297 SNPs, was as follows: Teno Escapees, 0.415; Finnmark Escapees, 0.348; Old Teno Mainstem, 0.381; New Teno Mainstem 0.376: Inarijoki, 0.340; Kevojoki, 0.377; Pulmankijarvi, 0.349; Tsarsjoki, 0.276; Utsjoki, 0.313; Naatamo, 0.300; Tornio, 0.280. Pairwise F_st_ values amongst the samples (All Escapee plus eight wild populations) are shown in Table 1. Mean pairwise F_st_ between aquaculture escapees and the six Teno populations was 0.097, compared to a mean pairwise F_st_ of 0.083 amongst the Teno populations. Relatively high pairwise F_st_ between Tsarsjoki and most other locations reflected the reduced genetic diversity in this population. With Tsarsjoki removed, mean aquaculture–wild pairwise F_st_ and mean amongst‐wild pairwise F_st_ were 0.084 and 0.067, respectively. The high pairwise F_st_ between Tornio and all the other populations reflected the phylogeographic distinctness of this population.

**Table 1:** Pairwise F_st_ between samples based on all 199,297 SNPs (above diagonal) and 200 discriminatory SNPS (below diagonal)

	All escapee	Inarijoki	Kevojoki	Pulmanki järvi	Teno Old Mainstem	Teno New Mainstem	Tsarsjoki	Utsjoki	Näätämö	Tornio
All escapee		0.078	0.091	0.094	0.054	0.056	0.163	0.103	0.095	0.170
Inarijoki	0.470		0.074	0.072	0.018	0.027	0.141	0.066	0.057	0.203
Kevojoki	0.466	0.087		0.076	0.057	0.078	0.114	0.069	0.131	0.217
Pulmankijärvi	0.471	0.096	0.084		0.062	0.074	0.170	0.105	0.111	0.220
Teno Old Mainstem	0.333	0.075	0.103	0.110		0.006	0.125	0.057	0.049	0.172
Teno New Mainstem	0.307	0.110	0.130	0.134	0.010		0.130	0.061	0.039	0.171
Tsarsjoki	0.529	0.141	0.090	0.140	0.161	0.191		0.034	0.162	0.288
Utsjoki	0.518	0.101	0.077	0.109	0.137	0.168	0.037		0.080	0.227
Näätämö	0.401	0.135	0.184	0.178	0.067	0.063	0.252	0.215		0.213
Tornio	0.272	0.417	0.410	0.424	0.259	0.232	0.487	0.471	0.319	

As expected, pairwise F_st_ between the All Escapee and wild populations was much higher when calculated only from the discriminatory subset of SNPs (Table S1, mean F_st_ across Teno/All Escapee comparisons = 0.464). Pairwise F_st_ values estimated between the New Teno Mainstem pools and the All Escapee sample were slightly higher when allele frequencies had been estimated by allelotyping than when they had been calculated by individual genotyping (Table S1, mean F_st_ from 199,297 loci = 0.067 vs. 0.058; mean F_st_ from 200 loci = 0.324 vs. 0.306).

### 3.4 Outlier analysis

In the comparison between all wild fish and all aquaculture escapees (All Wild/All Escapee), we found 227 outlying loci (*q* < .05) with Fdist2 and 1,112 (*q* < .05) with Bayescan, of which 183 overlapped between the two methodologies (Fig. S2; Table S2). Outlier loci were distributed across all 29 chromosomes (Fig. S2). More than 95% of these 1,156 loci were also identified as outliers in one or more of the comparisons between escapees and fish from different Teno locations (Kevojoki, Inarijoki, Pulmankijärvi, Tsarsjoki, Utsjoki or Old Teno Mainstem; 78% of loci were outliers in at least two comparisons, Table S2). Across the genome, 67 linked clusters of SNPs plus 60 single SNPs were identified by both Bayescan (*q* < .05) and Fdist2 (*q* < .05) as outliers in the All Wild/Escapee comparison (Table S2). The set of 200 SNPs selected for use in hybrid discrimination were distributed over 28 of the 29 Atlantic salmon chromosomes (Fig. S2, Tables S2, S3). None of these loci overlapped with those described by Karlsson et al. (2011). Estimated B‐allele frequencies for these SNPs and adjusted frequencies used for simulations are provided in Table S3. Pairwise F_st_ values between simulated escapee and wild populations at Generation 0 were lower than the unbiased pairwise F_st_ values calculated from estimated allele frequencies of the real samples (Table S1).

### 3.5 Assignment of simulated individuals to different hybrid classes

**Figure 3:**
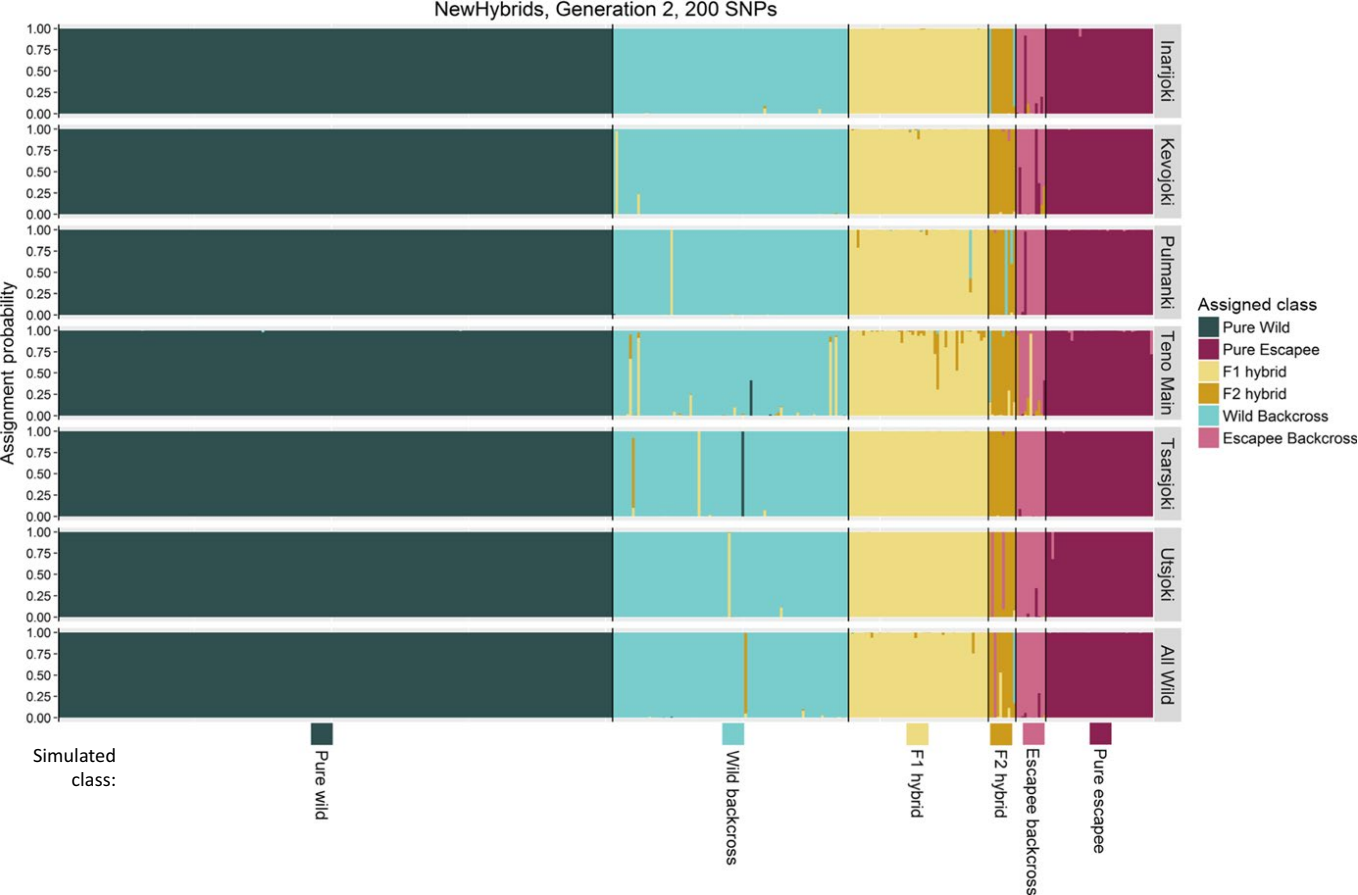
Results of NewHybrids analysis for 400 individuals produced by two generations of simulated hybridization between aquaculture escapees (10% of the population) and wild fish from different Teno subpopulations. Each individual is represented by a vertical bar. Individuals are arranged along the *x*‐axis by simulated hybrid class, with different hybrid classes bounded by black lines. *Y*‐axis indicates the probability, returned by New Hybrids, that an individual belongs to one of the six possible hybrid classes (‘Assignment probability’). The different possible hybrid classes are indicated by different colours. For ‘All Wild’, the wild population was simulated using the average allele frequencies over all seven subpopulations

Replicate runs of NewHybrids for the same data set gave almost identical outcomes, and in all cases, we present results from the first run. For the second generation of hybridization between wild fish and aquaculture fish (Generation 2), the full set of 200 SNPs exhibited a high *overall performance*, assigning all but 24 individuals (1%) to the correct hybrid class over all six simulated Teno subpopulations, irrespective of whether or not a reference sample was provided (Fig. 3, Fig. 5, Tables S4, S5). This performance barely changed when number of SNPs was reduced to 160 (Fig. 5, Fig. S3, Tables S4, S5: 1.4% misassignment). Further reductions in SNP number led to a decline in *overall performance*, particularly when assigning F2 hybrids and escapee backcrosses (Fig. 5, Fig. S3; Tables S4, S5). However, even using as few as 40 SNPs, only 12 hybrid individuals (all wild backcross, 1% of all hybrids) were erroneously identified as pure wild fish.

**Figure 4:**
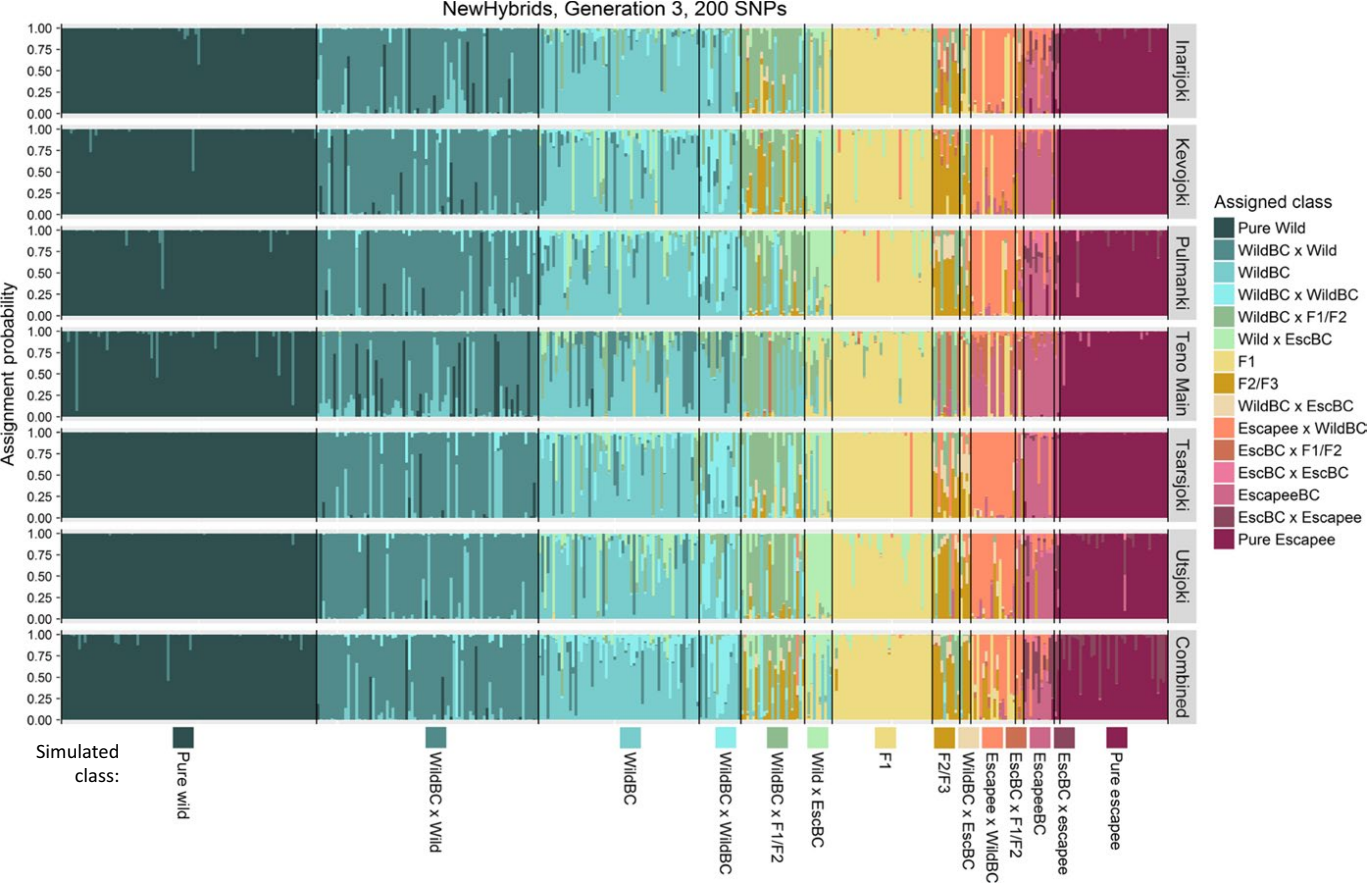
Results of NewHybrids analysis for 400 individuals produced by three generations of simulated hybridization between aquaculture escapees and wild fish. Fifteen different hybrid classes can potentially be discriminated by NewHybrids, of which one (EscBC X EscBC) is not present in the mixture due to low frequency. ‘X’: crossed with; ‘Esc’: escapee; ‘BC’: backcross; ‘F1/F2’: F1 hybrid or F2 hybrid; ‘F2/F3’: F2 hybrid or F3 hybrid. For further details see Fig. 3

**Figure 5:**
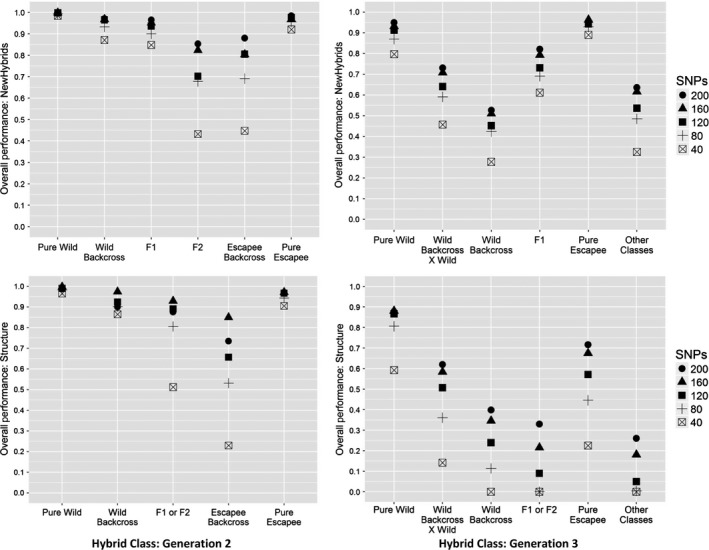
Overall performance of different numbers of SNPs for correctly assigning different hybrid classes generated by two or three generations of hybridization and using two different assignment methods. Top: NewHybrids; bottom: Structure

Assignment of individuals to the many possible hybrid classes created by three generations of wild–escapee hybridization (Generation 3) is a much more difficult analytical problem, and we focus our discussion on the five most frequent classes generated in our scenario of 10% escapees per generation: Pure Wild, Pure Escapee, F1 Hybrids, Wild Backcross and Wild Backcross X Wild. Using the full set of 200 SNPs, 99.1% of Pure Wild individuals, 96.4% of Pure Escapees, 94.8% of F1 Hybrids, 83.8% of Wild Backcross X Wild and 73.2% of Wild Backcross were assigned correctly (Fig. 4, Fig. 5, Table S6). However, rarer hybrid classes were correctly assigned with much lower success (Fig. 4, Table S6). Misassigned individuals were almost invariably assigned to hybrid classes with similar proportions of wild/escapee ancestry. As expected, performance declined with decreasing numbers of SNPs (Fig. 5, Fig S4, Table S6). Once again, misassignment of hybrid individuals to the Pure Wild class was rare and almost entirely limited to Wild Backcross X Wild individuals; even with 80 SNPs, only 4% of hybrids were incorrectly assigned as Pure Wild individuals.

Replicate runs of Structure for the same data set gave congruent results, and we report results from the first run. In general, Structure was a less effective analytical approach than NewHybrids for assigning simulated individuals to hybrid classes, particularly with fewer SNPs and after three generations of hybridization (Fig. 5, Figs S5, S6, Tables S7, S8). Nevertheless, at Generation 2, and with 120 or more SNPs, Structure and NewHybrids assigned individuals to the correct class with similar high levels of accuracy (Fig. 5, Fig. S5, Table S7).

### 3.6 Hybrid discrimination in additional wild populations

**Figure 6:**
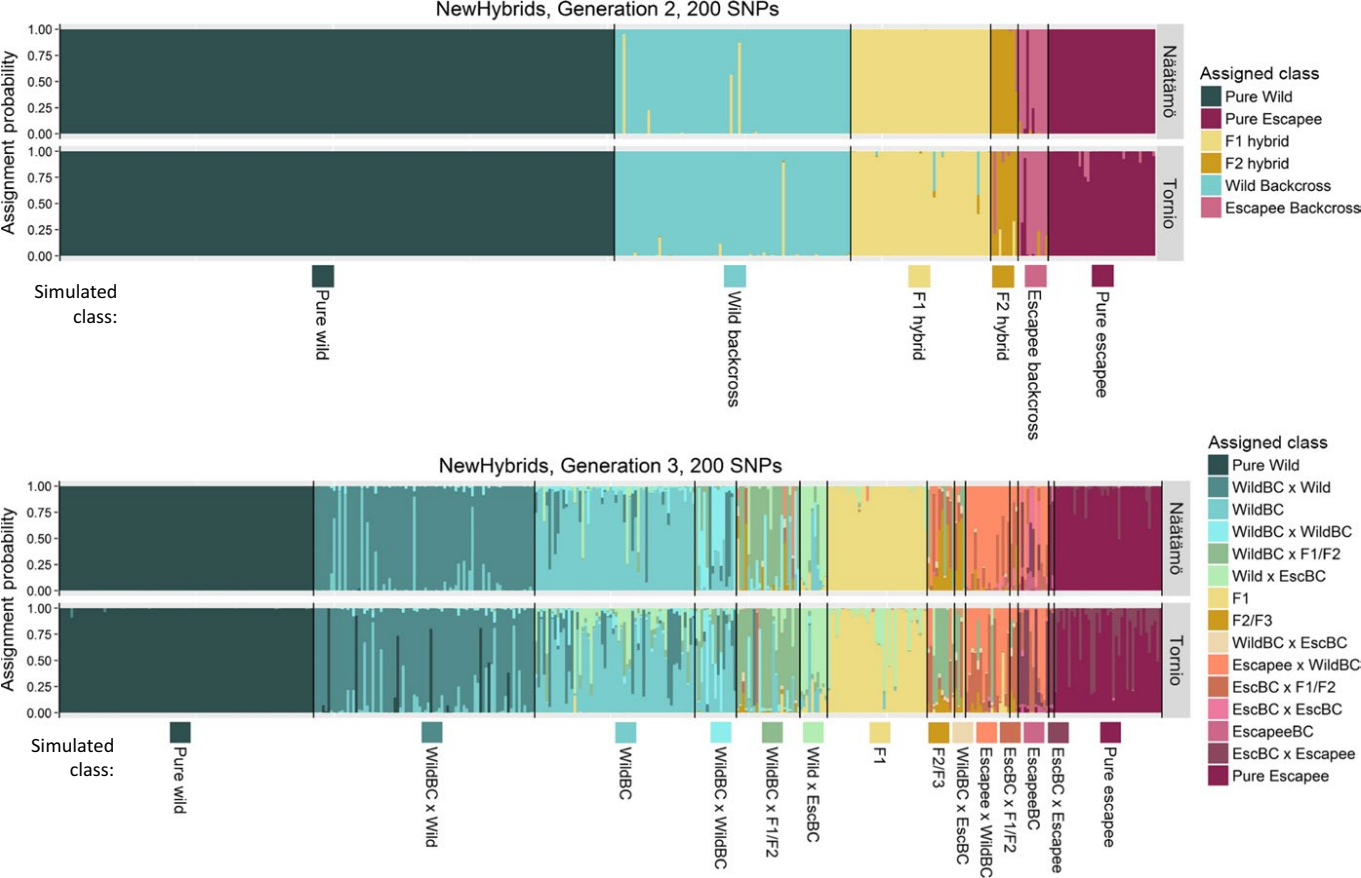
Results of NewHybrids analysis for 400 individuals produced by two and three generations of simulated hybridization between aquaculture escapees (10% of the population) and wild fish from Näätämö and Tornio. See Fig. 3 and Fig. 4 for further details

Simulated hybrids between aquaculture escapees and fish from Näätämö or Tornio were assigned to the correct hybrid class with similar accuracy to that observed in the simulations involving Teno populations (Fig. 6, Fig. S8, Tables S4, S5 & S6), especially using larger numbers of SNPs. Again, misassignment of simulated hybrids as pure wild fish was rare. With 200 SNPs, at Generation 2, there were no such misassignments for either Tornio or Näätämö. At Generation 3, no hybrids were misidentified as wild fish for Näätämö and <1.9% of hybrids were misidentified for Tornio, all of which were Wild Backcross X Wild.

## 4 Discussion

Here, we have shown that a suite of 200 SNPs can collectively discriminate advanced‐generation classes of hybrid between wild fish from a genetically diverse river and the aquaculture escapees that may be reproducing in that river. Our assessment of the efficacy of these markers is expected to be conservative because we deliberately simulated hybridizing populations with a lower level of genetic divergence between them than was estimated from our real population sample. For example, our simulated wild populations contained no monomorphic loci, while many of these loci may truly be monomorphic in several of the Teno subpopulations. For two generations of hybridization, these markers assign simulated individuals to their correct hybrid class with a very high level of accuracy. Even after three generations of hybridization, many hybrid classes can be reliably identified. Importantly, individuals with hybrid ancestry are rarely identified as pure wild fish in either of the hybrid generations examined. This is a much more accurate level of hybrid class identification than has previously been shown using genetic markers that discriminate aquaculture and wild fish (Karlsson et al., 2011). Further, we observe similarly good discrimination of hybrid classes when simulating hybrids from parental populations that were not originally used to identify the SNPs, including a population in a highly divergent evolutionary lineage, which suggests this marker set may be useful across a range of populations. The rather low number of 200 SNPs can nowadays be assayed relatively cheaply through genotyping via sequencing (Campbell, Harmon, & Narum, 2014) or similar approaches. Moreover, we have shown that a smaller subset of these SNPs enables hybrid class discrimination to a level of accuracy that may be sufficient in many scenarios.

To examine SNP allele frequencies in the majority of our populations, we used a cost‐effective allelotyping approach with the 220K Affymetrix SNP array. Comparison of allele frequencies estimated from individually genotyped fish and pools of the same individuals demonstrated that allelotyping, followed by the PPC correction, well approximated true allele frequencies. We note, however, that the PPC correction coefficients were largely estimated from the same set of samples that were subsequently used to test the accuracy of allele frequency estimates from allelotyping. The performance of these PPC correction coefficients in an independent set of samples has not yet been examined. In particular, in the sample used to generate the PPC correction, 2.3% of SNPs were missing one or more of the three possible genotypes, meaning that the coefficients for these SNPs were unlikely to be estimated correctly (Anantharaman & Chew, 2009). Further, the increased difficulty of estimating DNA concentration when it is partly degraded (Simbolo et al., 2013) may cause allelotyping accuracy to be lower for older scale samples because individuals contribute unequally to the pools. Nevertheless, many of the same outlier regions were identified when comparing the individually genotyped Old Teno Mainstem sample with the aquaculture escapees and when comparing the allelotyped Teno tributaries with the same escapees, suggesting that allelotyping did not bias the results of our analyses. Estimated levels of differentiation between All Escapee and New Teno Mainstem samples were higher when using allele frequencies estimated from allelotyping than when using those obtained from individual genotyping. This bias may partly be caused by rare shared alleles remaining undetected when allelotyping, and should be born in mind when combining results obtained from allelotyping and genotyping in other studies.

Utilizing escapees caught in the wild as our sample of aquaculture salmon enabled us both to capture a large range of aquaculture salmon genetic diversity (including variability over time), and directly sample individuals that were likely to be entering the spawning areas of the Teno River. Visualization of genomic differentiation amongst individually genotyped fish revealed a clear distinction between wild Teno Mainstem individuals (sampled both before and during aquaculture influence) and aquaculture escapees caught in the same river. Moreover, the aquaculture escapees fell into two distinct clusters, which were not strongly related to period of collection, and are likely to represent different aquaculture lines. Norwegian Atlantic salmon aquaculture strains have mixed ancestry from multiple wild populations and are known to be genetically diverse (Glover, Hansen, & Skaala, 2009; Karlsson et al., 2011). This is reflected in the relatively high heterozygosity that we observe within our aquaculture escapee sample, although estimated H_e_ is also expected to be biased higher in escapees due to the preferential inclusion of SNPs that are polymorphic in aquaculture lines in the 220K array. Previous microsatellite genotyping of the Teno Escapees also found them to be genetically diverse (Erkinaro et al., 2010).

We selected discriminatory SNPs from regions of the genome that are unusually divergent between wild Teno River salmon and aquaculture escapees. This F_st_ outlier approach has previously been used successfully to identify SNPs for stock discrimination in *Oncorhynchus nerka* (Ackerman, Habicht, & Seeb, 2011; Russello, Kirk, Frazer, & Askey, 2012) and wild *S. salar* (Ozerov et al., 2013). Outlying regions are expected to harbour loci under differential selection between the studied groups. Some of the regions that we identified here may contain loci under convergent directional selection in aquaculture strains compared to wild fish. However, given that Norwegian aquaculture strains are primarily derived from a different evolutionary lineage to Teno salmon (Bourret et al., 2013), it is likely that a proportion of these outlying regions reflect long‐term evolutionary divergence between the Atlantic (escapee) and North Barents/White Sea (Teno) lineages. Disentangling these two sources of genomic variation between wild Teno fish and aquaculture escapees, and identifying the genes potentially under selection, requires assessment of additional samples from populations in the Atlantic and North Barents/White Sea lineages and is outside the realm of this study.

The fact that the 220K Affymetrix SNP array that we used is optimized for SNPs variable within Norwegian aquaculture lines limited our ability to identify truly discriminatory markers. Ninety per cent of our 200 ‘discriminatory’ SNPs had minor allele frequencies >0.3 in the All Escapee sample. In contrast, 93% of these SNPs had minor allele frequencies <0.1 in the All Wild sample (Table S3). Identification of a set of SNPs with fixed differences between wild salmon and aquaculture lines would greatly increase the accuracy of later generation hybrid identification, with fewer markers being required (Vähä & Primmer, 2006). The outlying genomic regions that we have identified with the genome scan approach are likely to be enriched for such diagnostic SNPs, and targeted sequencing of these regions is a promising approach to find such markers in the future.

We expect the set of 200 SNPs that we have identified in this study to be useful in investigating wild–aquaculture introgression in all Atlantic salmon belonging to the North Barents/White Sea evolutionary lineage, which includes important populations in northern Norway, Finland and Russia (Bourret et al. 2013). Farming of Norwegian aquaculture salmon is continuing to expand in this region, particularly in Russia (ICES 2015), increasing the introgression threat to these northern populations. This set of 200 SNPs also proved effective at discriminating different classes of simulated hybrids between aquaculture escapees and wild salmon from the Tornio River, which is within the highly divergent Baltic evolutionary lineage (Bourret et al. 2013). Examination of allele frequencies within our samples (Table S3) suggests that our SNP selection procedure has pinpointed many loci with alleles at relatively high frequency within Norwegian aquaculture lines but at low frequency or absent in other lineages. Baltic Atlantic salmon populations are not currently at risk for hybridization with aquaculture escapees, due to environmental conditions (including the presence of the parasite *Gyrodactylus salaris*, Zueva et al., 2014) that are unsuitable for commercial aquaculture strains. However, the strong performance of our SNP set in discriminating different classes of simulated hybrid between aquaculture escapees and the Tornio population suggests they might perform similarly well in other regions where the native wild Atlantic salmon populations are genetically distinct from the Norwegian ancestors of the aquaculture lines. Although several countries have developed their own aquaculture lines from local salmon populations (e.g. Bourret et al., 2011), Norwegian aquaculture strains are also utilized worldwide (e.g. Clifford et al., 1998).

Even if the set of SNPs described here do not perform well in other regions, our results suggest that, using our approach, it may be straightforward to find similar markers that identify hybrids between Norwegian aquaculture strains and other genetically divergent populations. This will be facilitated by the recent development of several other high‐density Atlantic salmon SNP arrays (Houston et al., 2014; Yáñez, Houston, & Newman, 2014). Identifying SNPs to discriminate Norwegian aquaculture strains and the wild central and southern Norwegian populations from which they are largely derived is expected to be a more difficult task. The work of Karlsson et al. (2011) was directed at finding markers to study the important problem of wild–escapee hybridization within this evolutionary lineage. Although the Karlsson et al. (2011) SNPs have not been tested for their usefulness at discriminating hybrids beyond the F1, they are expected to be less effective for the identification of later generation hybrid classes simply because of the much lower genetic divergence between the hybridizing lineages compared to our study. The genome scan approach that we have applied here provides opportunities to identify additional discriminatory markers in genomic regions that have diverged between aquaculture fish and their wild ancestors due to shared directional selection amongst domestic lines.

In summary, we have demonstrated that a set of 200 SNPs enables discrimination of different classes of hybrid between wild Atlantic salmon and aquaculture escapees for up to three generations of hybridization. This suite of SNPs will allow detailed examination of the hybridization dynamics between aquaculture escapees and native fish in an important wild Atlantic salmon population. We expect these SNPs, and/or the analytical approaches that we have used to identify them, to also be useful when investigating escapee–wild hybridization throughout a much wider geographic range.

## Data Archiving Statement

Raw data and code used in analyses are archived in the Dryad Digital Repository: http://dx.doi.org/10.5061/dryad.dg8f3.
