# Supplementary material for: Single nucleotide polymorphisms to discriminate different classes of hybrid between wild Atlantic salmon and aquaculture escapees
Source: Evol Appl. 2016 Aug 18;9(8):1017–31. doi: 10.1111/eva.12407 (PMC4999531; doi:10.1111/eva.12407)
Supplement: Supplementary file 1 [file EVA-9-1017-s001.pdf]

SNPs to discriminate different classes of hybrid between wild Atlantic salmon and aquaculture escapees: Supplementary Figures.

Figure S1: Relationship between true B allele frequency obtained through individual genotyping, and B allele frequency estimated by allelotyping, for the four New Teno Mainstem pools. Top: raw estimated frequencies before PPC correction, 199,297 SNPs; middle: estimated frequencies following PPC correction, 199,297 SNPs; bottom: 200 SNPs used for hybrid discrimination.

Figure S1

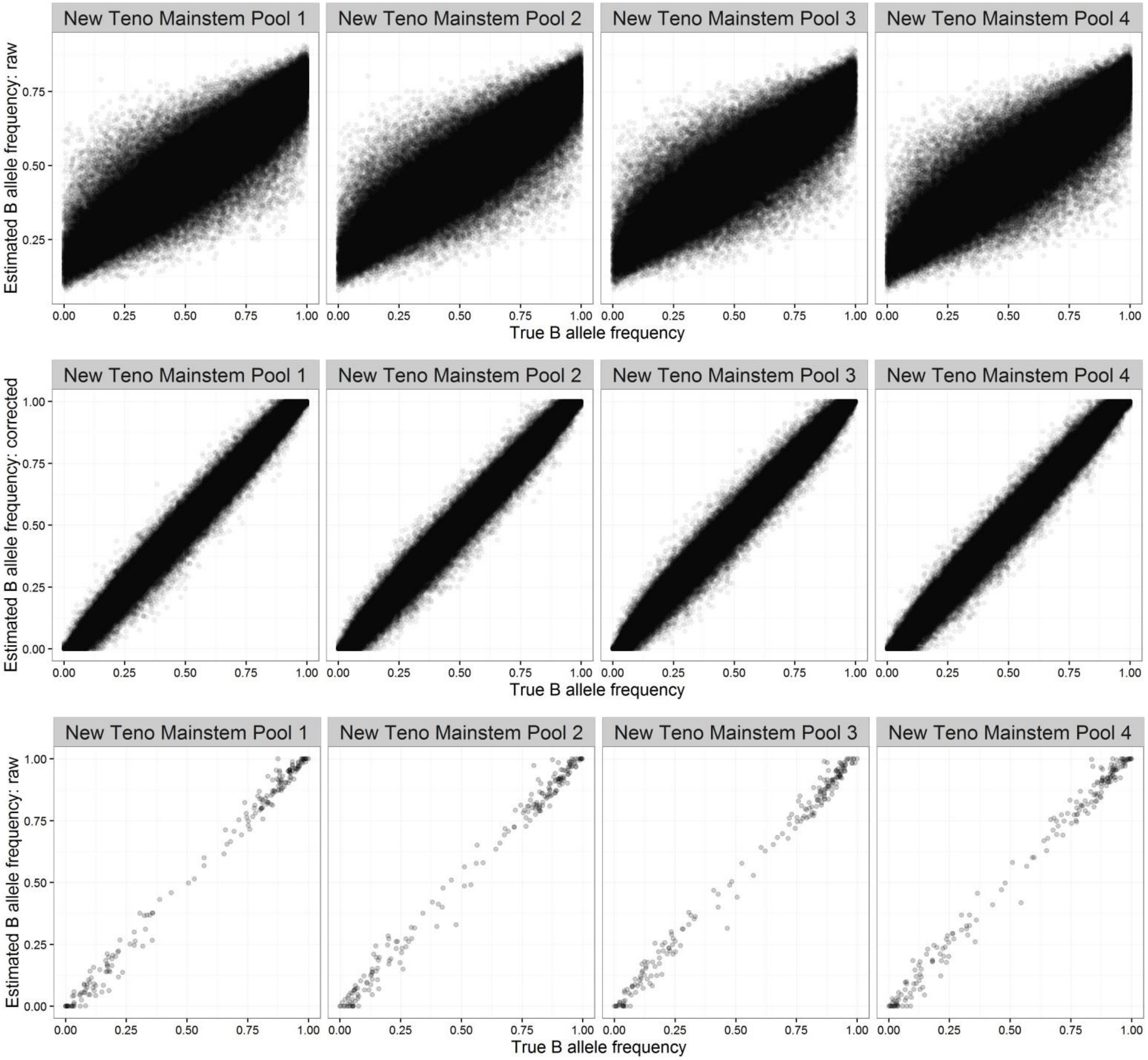

New Teno Mainstem Pool 1
New Teno Mainstem Pool 2
New Teno Mainstem Pool 3
New Teno Mainstem Pool 4
Estimated B allele frequency: raw
Estimated B allele frequency: corrected
True B allele frequency
0.00
0.25
0.50
0.75
1.00
