# Supplementary material for: Single nucleotide polymorphisms to discriminate different classes of hybrid between wild Atlantic salmon and aquaculture escapees
Source: Evol Appl. 2016 Aug 18;9(8):1017–31. doi: 10.1111/eva.12407 (PMC4999531; doi:10.1111/eva.12407)
Supplement: Supplementary file 2 [file EVA-9-1017-s002.pdf]

SNPs to discriminate different classes of hybrid between wild Atlantic salmon and aquaculture escapees: Supplementary Figures.

Figure S2: Significance of F_{st} outliers for the comparison between the combined wild fish and combined aquaculture escapee samples. Outliers were identified using two different approaches (Bayescan & Fdist2). The 199,297 SNPs are ordered by position on the genome, with chromosome number indicated along the x axis. Black dotted lines and red dotted line indicate a q value of 0.05, 0.01 and 0.0001 respectively. Q values of zero were converted to 0.000005 to allow representation on a –log10 scale. The 200 SNPs used for hybrid class discrimination are circled in red.

Figure S2

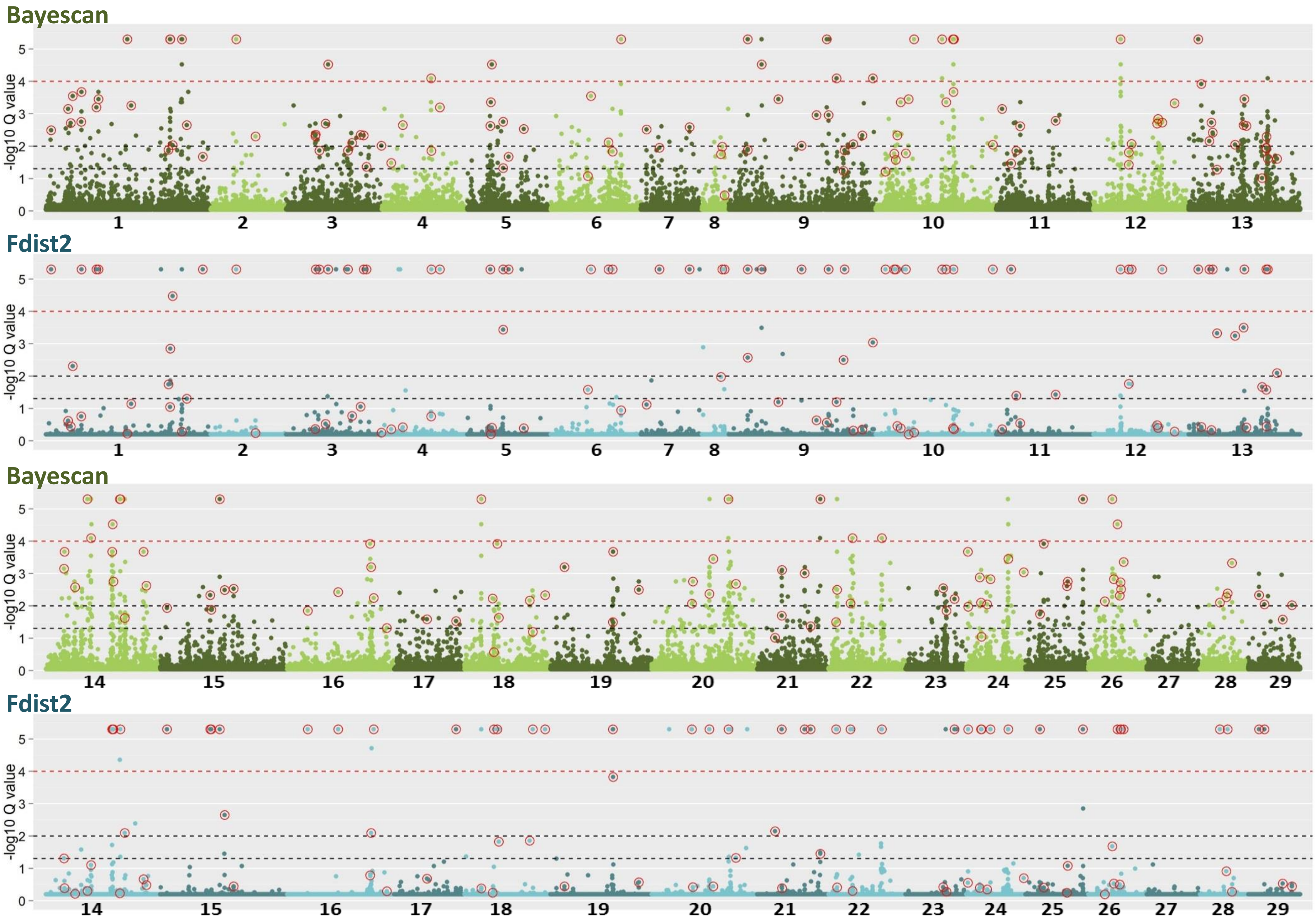

Bayescan
-log10 Q value
1 2 3 4 5 6 7 8 9 10 11 12 13
Fdist2
-log10 Q value
1 2 3 4 5 6 7 8 9 10 11 12 13
Bayescan
-log10 Q value
14 15 16 17 18 19 20 21 22 23 24 25 26 27 28 29
Fdist2
-log10 Q value
14 15 16 17 18 19 20 21 22 23 24 25 26 27 28 29
