# Supplementary material for: Single nucleotide polymorphisms to discriminate different classes of hybrid between wild Atlantic salmon and aquaculture escapees
Source: Evol Appl. 2016 Aug 18;9(8):1017–31. doi: 10.1111/eva.12407 (PMC4999531; doi:10.1111/eva.12407)
Supplement: Supplementary file 3 [file EVA-9-1017-s003.pdf]

SNPs to discriminate different classes of hybrid between wild Atlantic salmon and aquaculture escapees: Supplementary Figures.

Figure S3 (a-d) Results of NewHybrids analyses for 400 individuals produced by two generations of simulated hybridization between aquaculture escapees (10% of the population) and wild fish from six different Teno sub-populations. Each individual is represented by a vertical bar. Individuals are arranged along the x axis by simulated hybrid class, with different hybrid classes bounded by black lines. Y axis indicates the probability, returned by New Hybrids, that an individual belongs to one of the six possible hybrid classes ('Assignment probability'). The different possible hybrid classes are indicated by different colours. Different analyses are based on different number of SNPs.

Figure S4 (a-d) Results of NewHybrids analyses for 400 individuals produced by three generations of simulated hybridization between aquaculture escapees and wild fish. Fifteen different hybrid classes can potentially be discriminated by NewHybrids, of which one (EscBC_X_EscBC) is not present in the mixture due to low frequency. 'Esc': escapee; 'BC': backcross. For further details see Figure S3.

Figure S3a

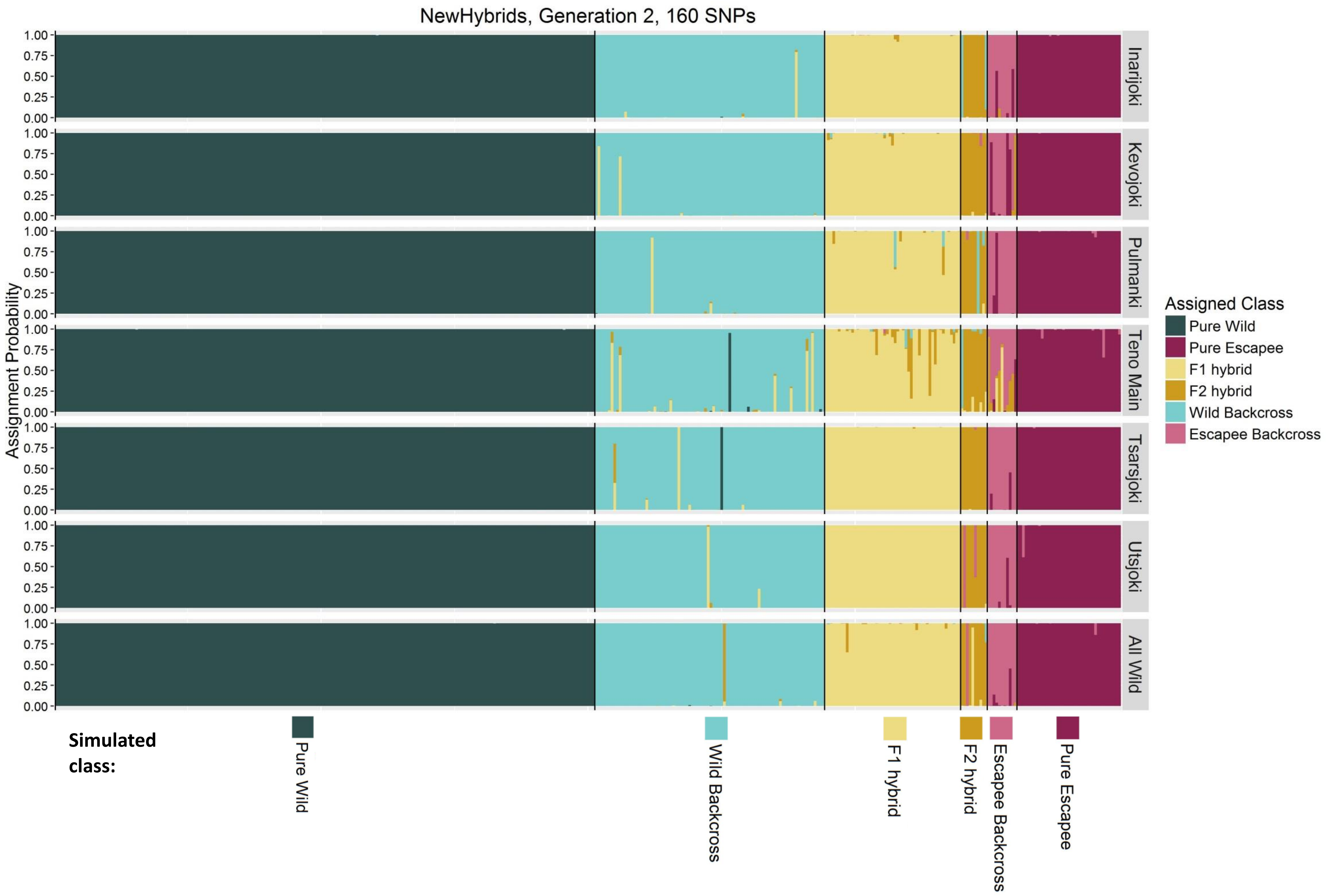
NewHybrids, Generation 2, 160 SNPs
Assignment Probability
1.00
0.75
0.50
0.25
0.00
Inarijoki
Kevojoki
Pulmanki
Teno Main
Tsarsjoki
Utsjoki
All Wild
Assigned Class
Pure Wild
Pure Escapee
F1 hybrid
F2 hybrid
Wild Backcross
Escapee Backcross
Simulated class:
Pure Wild
Wild Backcross
F1 hybrid
F2 hybrid
Escapee Backcross
Pure Escapee

Figure S3b

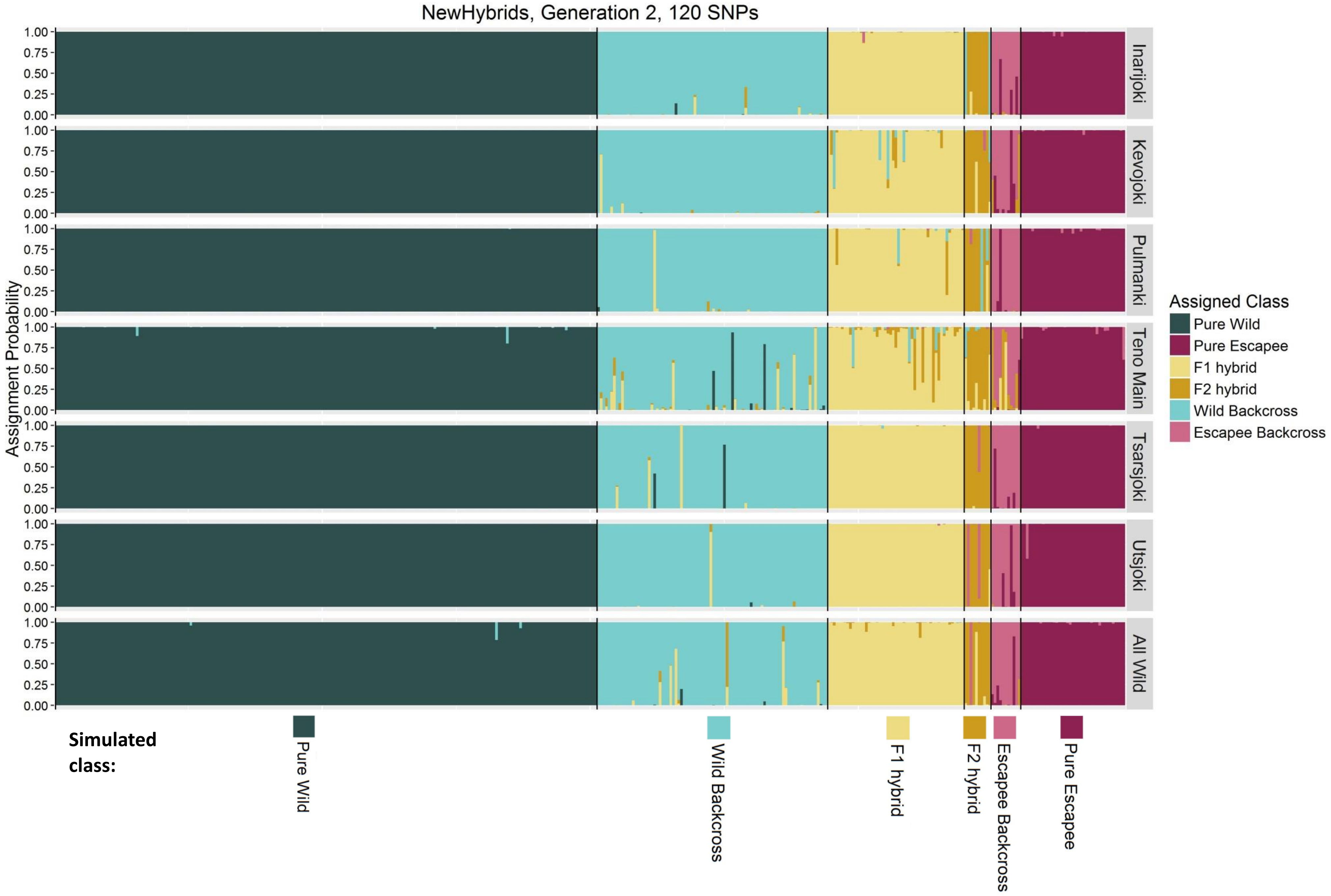
NewHybrids, Generation 2, 120 SNPs
Assignment Probability
1.00
0.75
0.50
0.25
0.00
Inarijoki
Kevojoki
Pulmanki
Teno Main
Tsarsjoki
Utsjoki
All Wild
Assigned Class
Pure Wild
Pure Escapee
F1 hybrid
F2 hybrid
Wild Backcross
Escapee Backcross
Simulated class:
Pure Wild
Wild Backcross
F1 hybrid
F2 hybrid
Escapee Backcross
Pure Escapee

Figure S3c

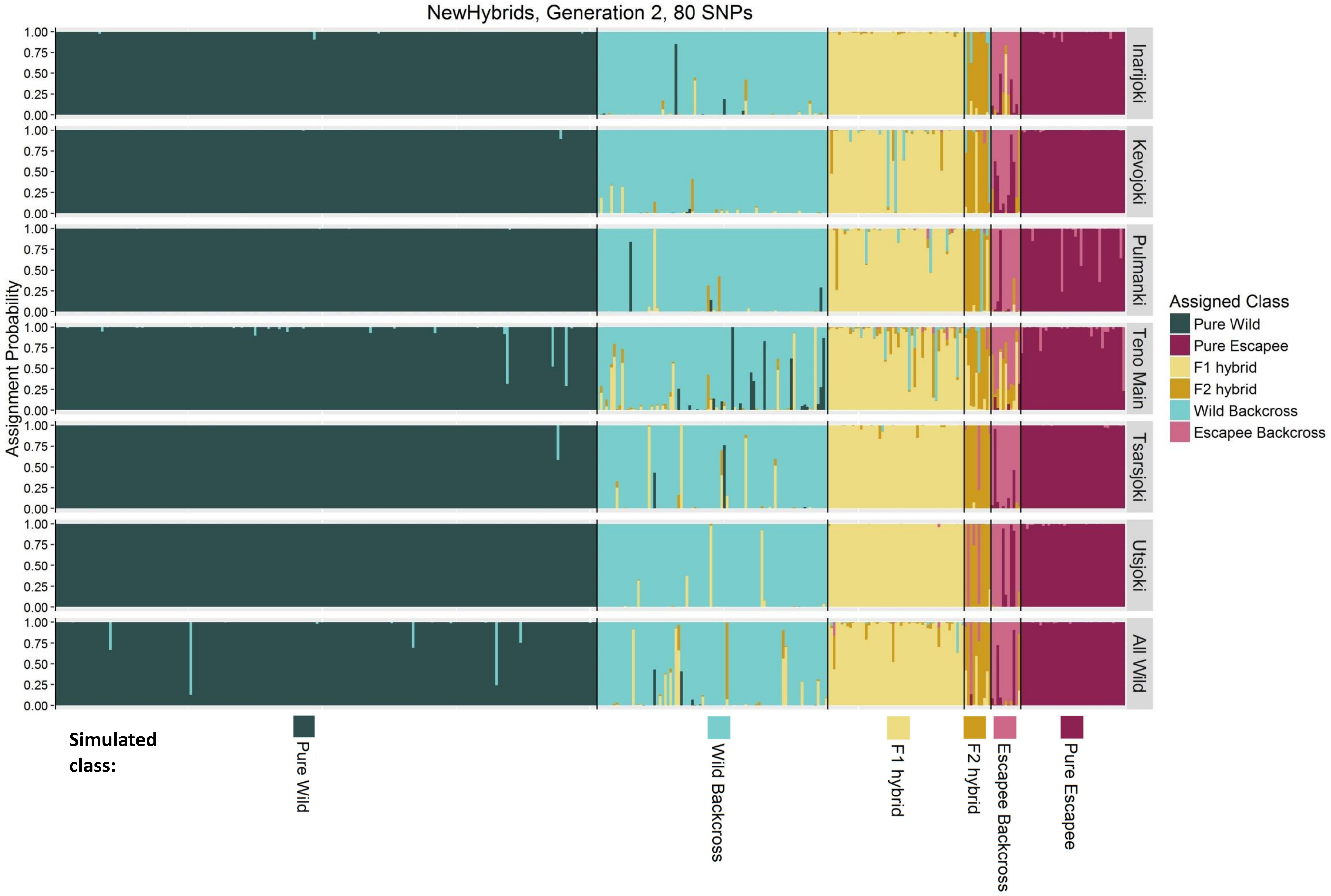
NewHybrids, Generation 2, 80 SNPs
Assignment Probability
1.00
0.75
0.50
0.25
0.00
Inarijoki
Kevojoki
Pulmanki
Teno Main
Tsarsjoki
Utsjoki
All Wild
Assigned Class
Pure Wild
Pure Escapee
F1 hybrid
F2 hybrid
Wild Backcross
Escapee Backcross
Simulated class:
Pure Wild
Wild Backcross
F1 hybrid
F2 hybrid
Escapee Backcross
Pure Escapee

Figure S3d

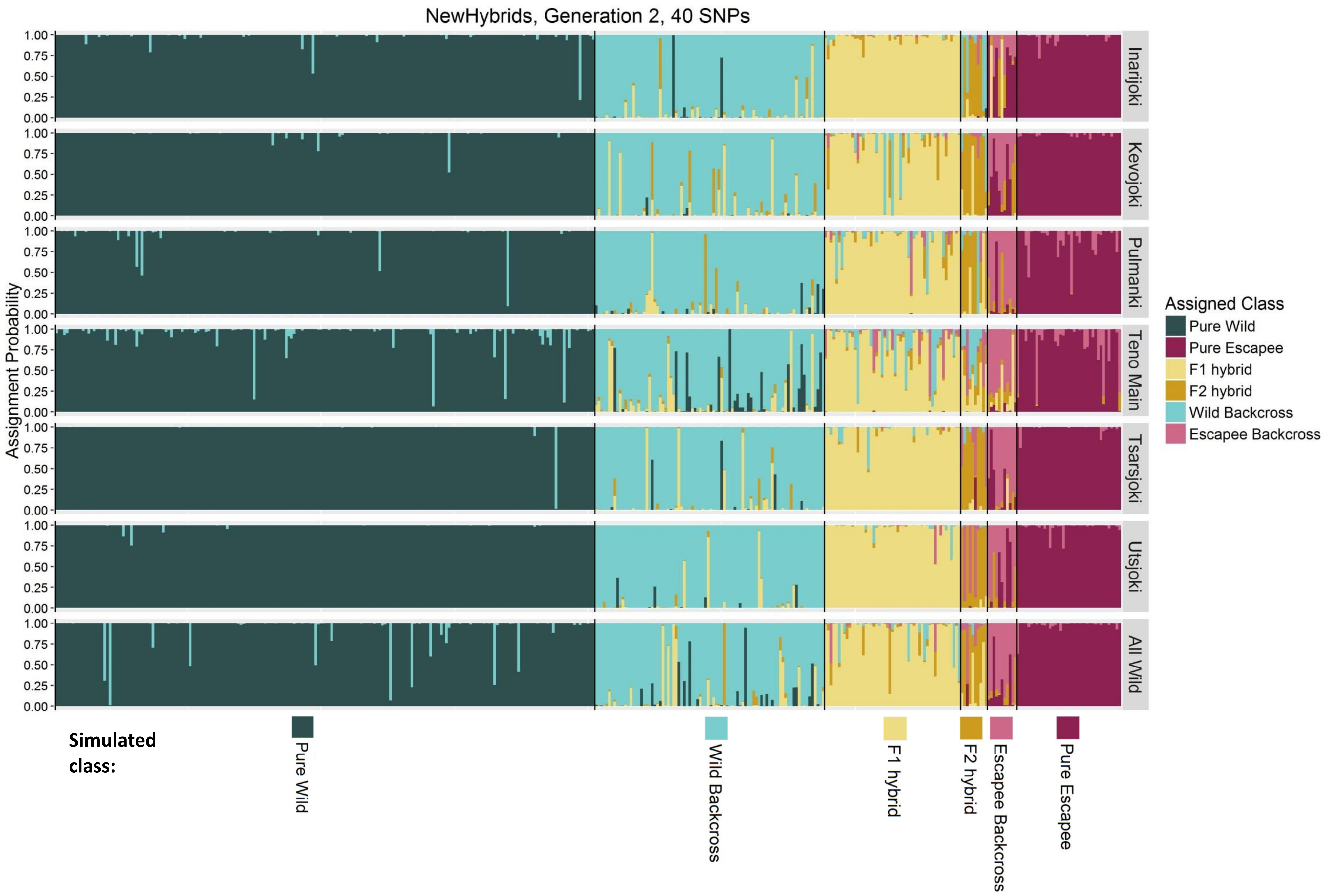

NewHybrids, Generation 2, 40 SNPs
Assignment Probability
1.00
0.75
0.50
0.25
0.00
Inarijoki
Kevojoki
Pulmanki
Teno Main
Tsarsjoki
Utsjoki
All Wild
Assigned Class
Pure Wild
Pure Escapee
F1 hybrid
F2 hybrid
Wild Backcross
Escapee Backcross
Simulated class:
Pure Wild
Wild Backcross
F1 hybrid
F2 hybrid
Escapee Backcross
Pure Escapee

Figure S4a

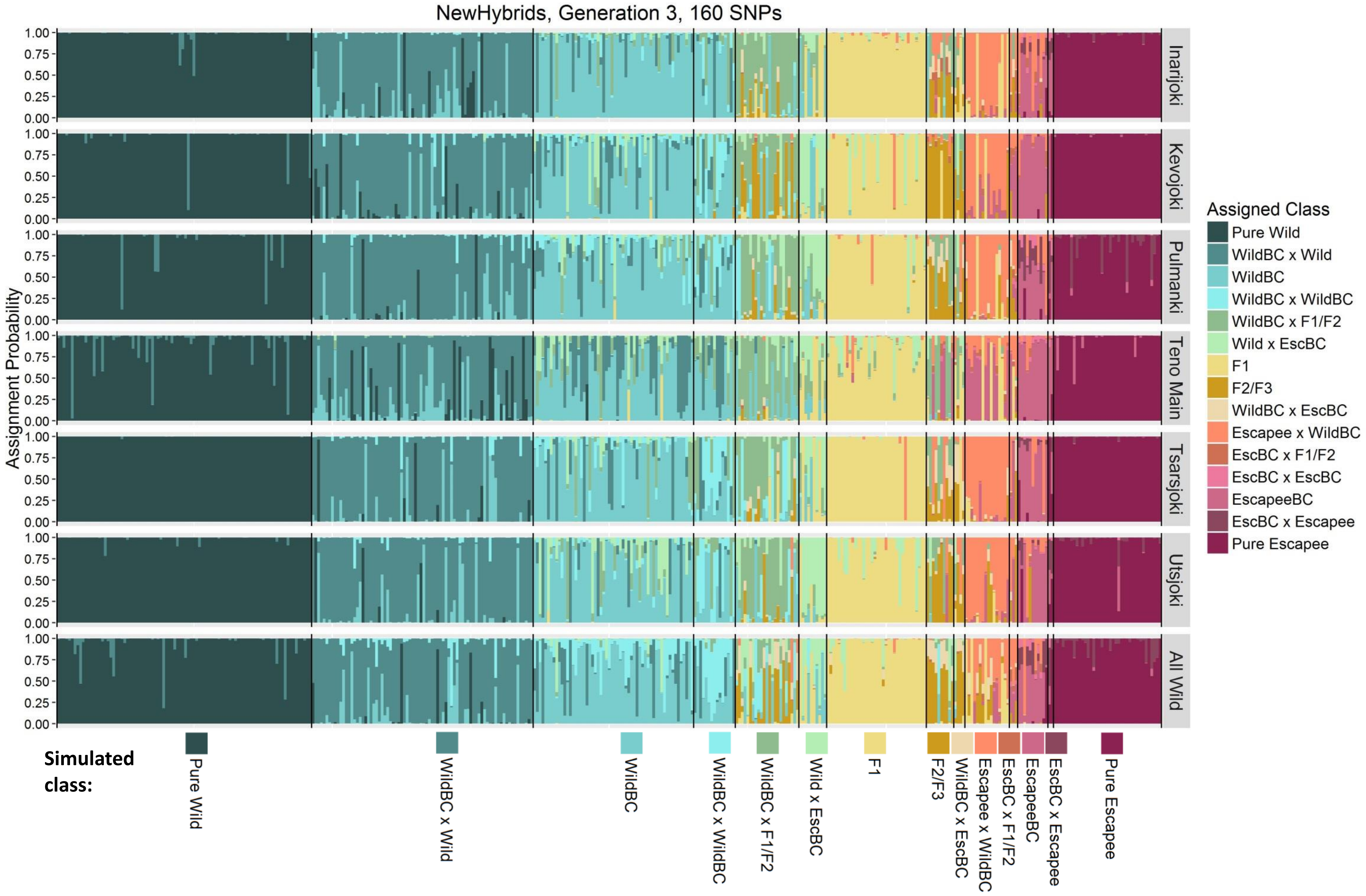
NewHybrids, Generation 3, 160 SNPs
Assignment Probability
1.00
0.75
0.50
0.25
0.00
Inarijoki
Kevojoki
Pulmanki
Teno Main
Tsarsjoki
Utsjoki
All Wild
Assigned Class
Pure Wild
WildBC x Wild
WildBC
WildBC x WildBC
WildBC x F1/F2
Wild x EscBC
F1
F2/F3
WildBC x EscBC
Escapee x WildBC
EscBC x F1/F2
EscBC x EscBC
EscapeeBC
EscBC x Escapee
Pure Escapee
Simulated class:
Pure Wild
WildBC x Wild
WildBC
WildBC x WildBC
WildBC x F1/F2
Wild x EscBC
F1
F2/F3
WildBC x EscBC
Escapee x WildBC
EscBC x F1/F2
EscapeeBC
EscBC x Escapee
Pure Escapee

Figure S4b

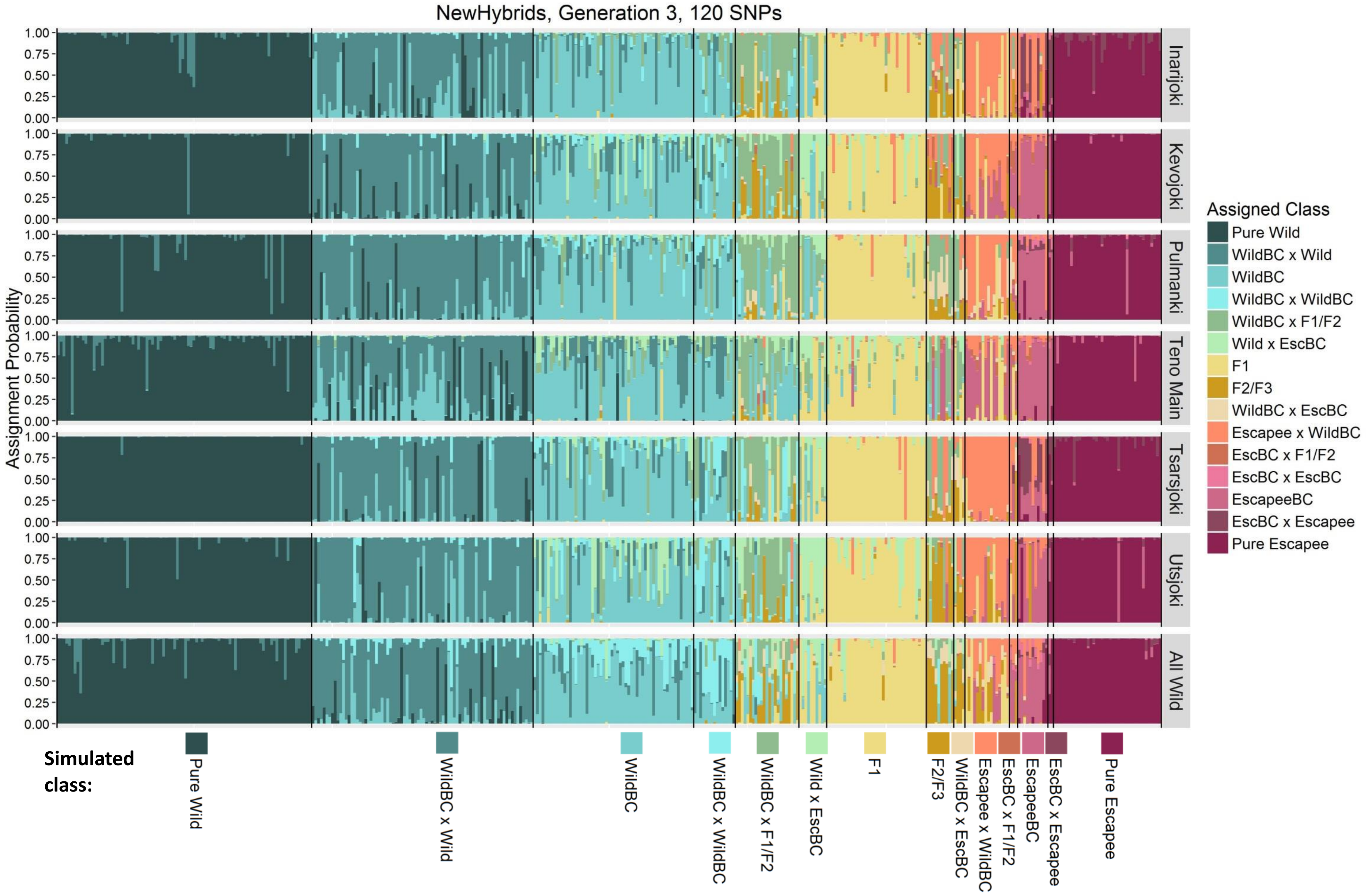
NewHybrids, Generation 3, 120 SNPs
Assignment Probability
0.00
0.25
0.50
0.75
1.00
Inarijoki
Kevojoki
Pulmanki
Teno Main
Tsarsjoki
Utsjoki
All Wild
Assigned Class
Pure Wild
WildBC x Wild
WildBC
WildBC x WildBC
WildBC x F1/F2
Wild x EscBC
F1
F2/F3
WildBC x EscBC
Escapee x WildBC
EscBC x F1/F2
EscBC x EscBC
EscapeeBC
EscBC x Escapee
Pure Escapee
Simulated class:
Pure Wild
WildBC x Wild
WildBC
WildBC x WildBC
WildBC x F1/F2
Wild x EscBC
F1
F2/F3
WildBC x EscBC
Escapee x WildBC
EscBC x F1/F2
EscapeeBC
EscBC x Escapee
Pure Escapee

Figure S4c

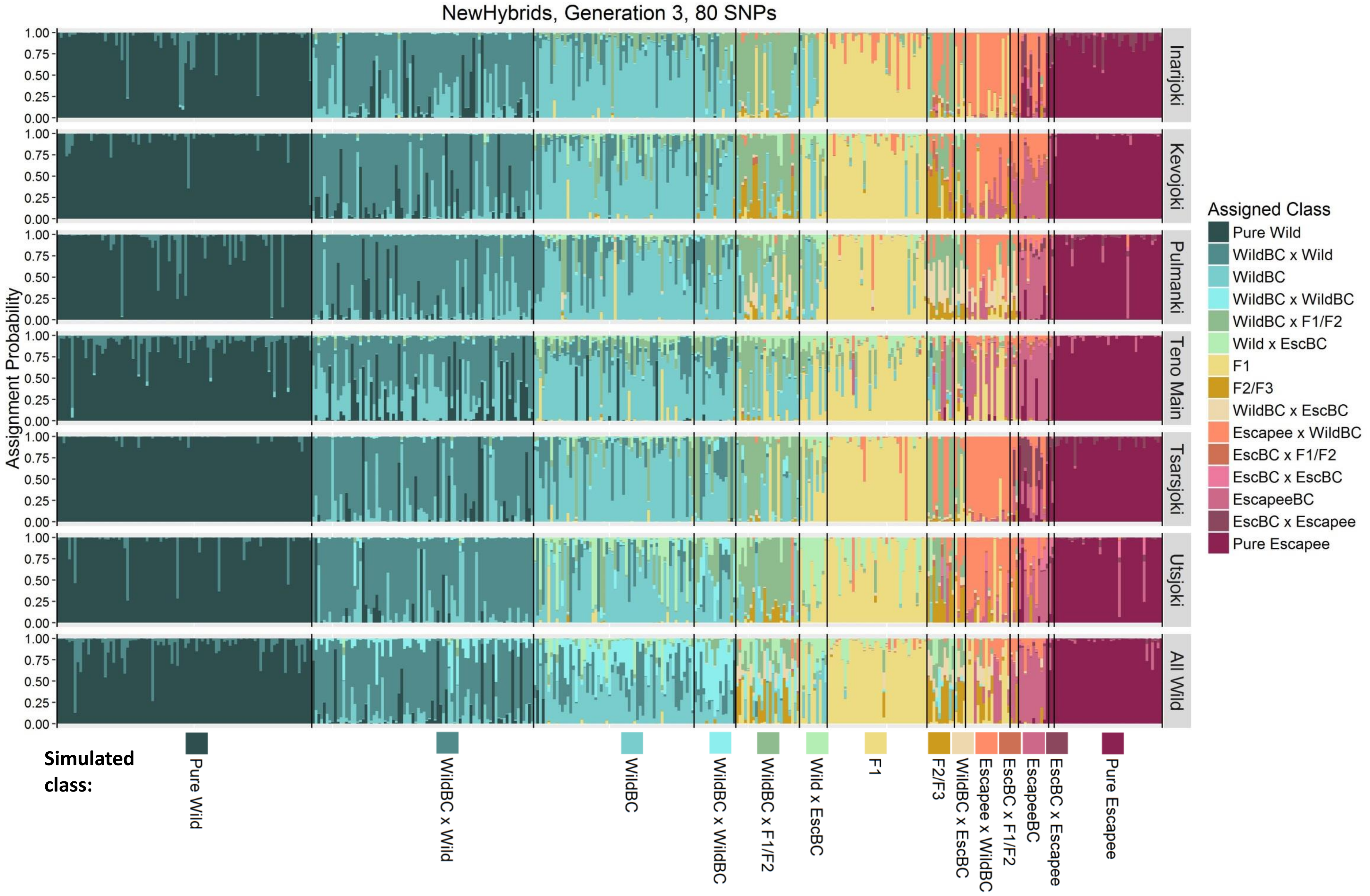

NewHybrids, Generation 3, 80 SNPs
Assignment Probability
1.00
0.75
0.50
0.25
0.00
Inarijoki
Kevojoki
Pulmanki
Teno Main
Tsarsjoki
Utsjoki
All Wild
Assigned Class
Pure Wild
WildBC x Wild
WildBC
WildBC x WildBC
WildBC x F1/F2
Wild x EscBC
F1
F2/F3
WildBC x EscBC
Escapee x WildBC
EscBC x F1/F2
EscBC x EscBC
EscapeeBC
EscBC x Escapee
Pure Escapee
Simulated class:
Pure Wild
WildBC x Wild
WildBC
WildBC x WildBC
WildBC x F1/F2
Wild x EscBC
F1
F2/F3
WildBC x EscBC
Escapee x WildBC
EscBC x F1/F2
EscapeeBC
EscBC x Escapee
Pure Escapee

Figure S4d

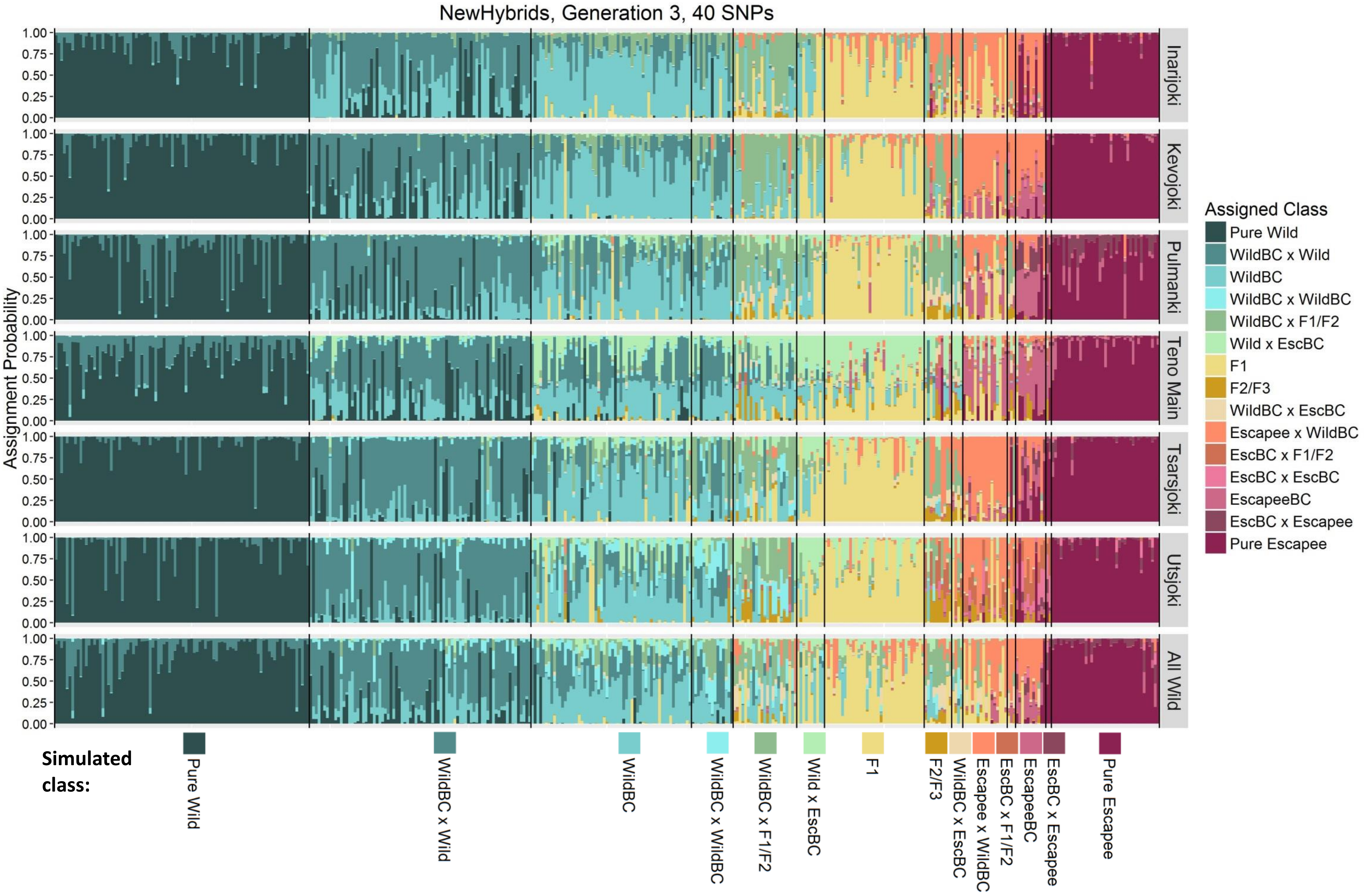
NewHybrids, Generation 3, 40 SNPs
Assignment Probability
1.00
0.75
0.50
0.25
0.00
Inarijoki
Kevojoki
Pulmanki
Teno Main
Tsarsjoki
Utsjoki
All Wild
Assigned Class
Pure Wild
WildBC x Wild
WildBC
WildBC x WildBC
WildBC x F1/F2
Wild x EscBC
F1
F2/F3
WildBC x EscBC
Escapee x WildBC
EscBC x F1/F2
EscBC x EscBC
EscapeeBC
EscBC x Escapee
Pure Escapee
Simulated class:
Pure Wild
WildBC x Wild
WildBC
WildBC x WildBC
WildBC x F1/F2
Wild x EscBC
F1
F2/F3
WildBC x EscBC
Escapee x WildBC
EscBC x F1/F2
EscapeeBC
EscBC x Escapee
Pure Escapee
