# Supplementary material for: Single nucleotide polymorphisms to discriminate different classes of hybrid between wild Atlantic salmon and aquaculture escapees
Source: Evol Appl. 2016 Aug 18;9(8):1017–31. doi: 10.1111/eva.12407 (PMC4999531; doi:10.1111/eva.12407)
Supplement: Supplementary file 4 [file EVA-9-1017-s004.pdf]

SNPs to discriminate different classes of hybrid between wild Atlantic salmon and aquaculture escapees: Supplementary Figures.

Figure S5 (a-e): Results of Structure analyses for 400 individuals produced by two generations of simulated hybridization between aquaculture escapees (10% of the population) and wild fish from six different Teno sub-populations. Each point represents an individual. Y axis indicates the estimated proportion of wild ancestry (± 95% confidence intervals) for each simulated individual. Colour of points indicates the actual hybrid class of each individual. Different analyses are based on different numbers of SNPs.

Figure S6 (a-e): Results of Structure analysis for 400 individuals produced by three generations of simulated hybridization. 'Esc': escapee; 'BC': backcross. For further details see Figure S4.

Figure S5a

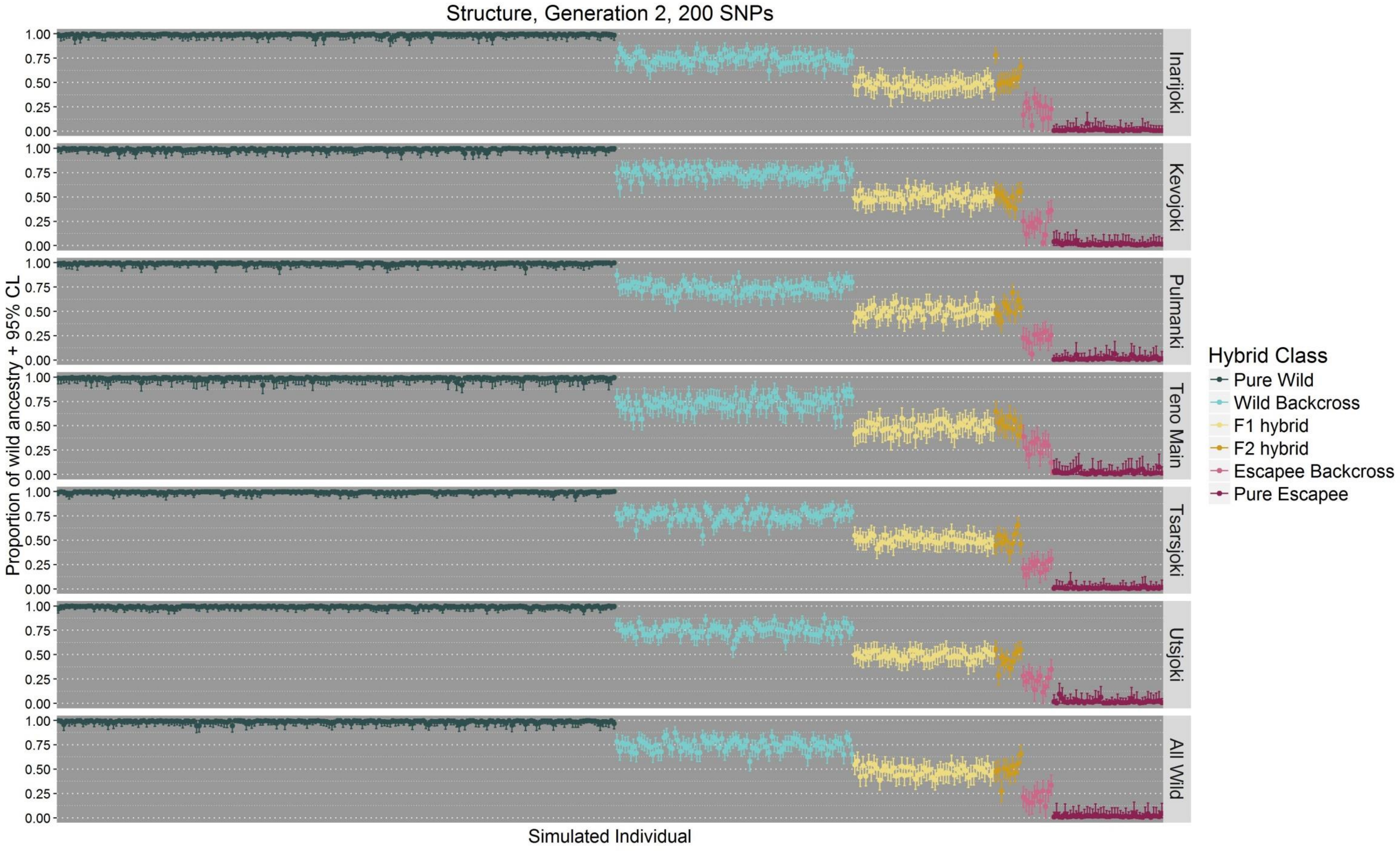
Structure, Generation 2, 200 SNPs
Proportion of wild ancestry + 95% CL
1.00
0.75
0.50
0.25
0.00
Inarijoki
Kevojoki
Pulmanki
Teno Main
Tsarsjoki
Utsjoki
All Wild
Simulated Individual
Hybrid Class
Pure Wild
Wild Backcross
F1 hybrid
F2 hybrid
Escapee Backcross
Pure Escapee

Figure S5b

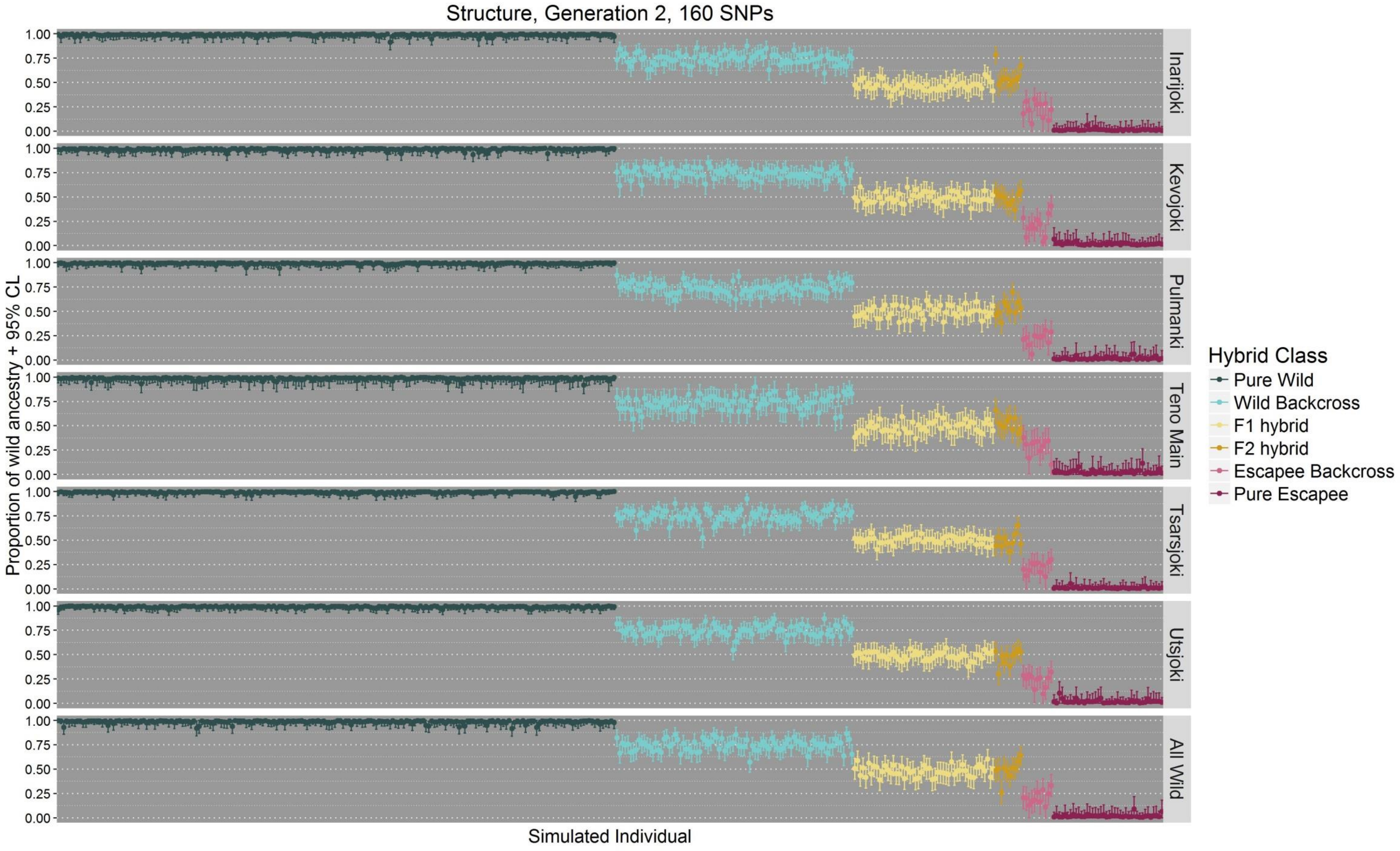

Structure, Generation 2, 160 SNPs
1.00
0.75
0.50
0.25
0.00
Proportion of wild ancestry + 95% CL
Inarijoki
Kevojoki
Pulmanki
Teno Main
Tsarsjoki
Utsjoki
All Wild
Simulated Individual
Hybrid Class
Pure Wild
Wild Backcross
F1 hybrid
F2 hybrid
Escapee Backcross
Pure Escapee

Figure S5c

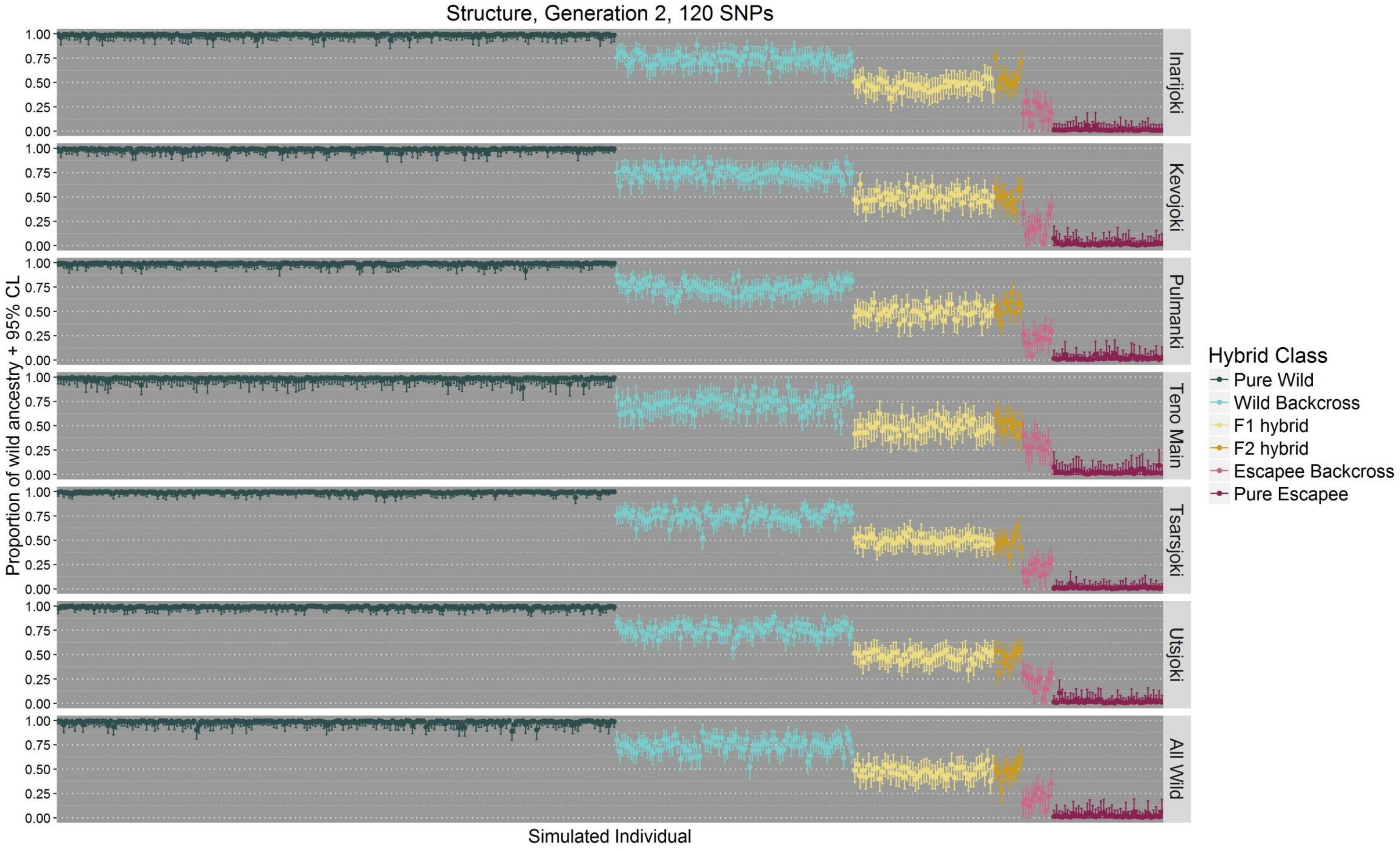
Structure, Generation 2, 120 SNPs
Proportion of wild ancestry + 95% CL
1.00
0.75
0.50
0.25
0.00
Inarijoki
Kevojoki
Pulmanki
Teno Main
Tsarsjoki
Utsjoki
All Wild
Simulated Individual
Hybrid Class
Pure Wild
Wild Backcross
F1 hybrid
F2 hybrid
Escapee Backcross
Pure Escapee

Figure S5d

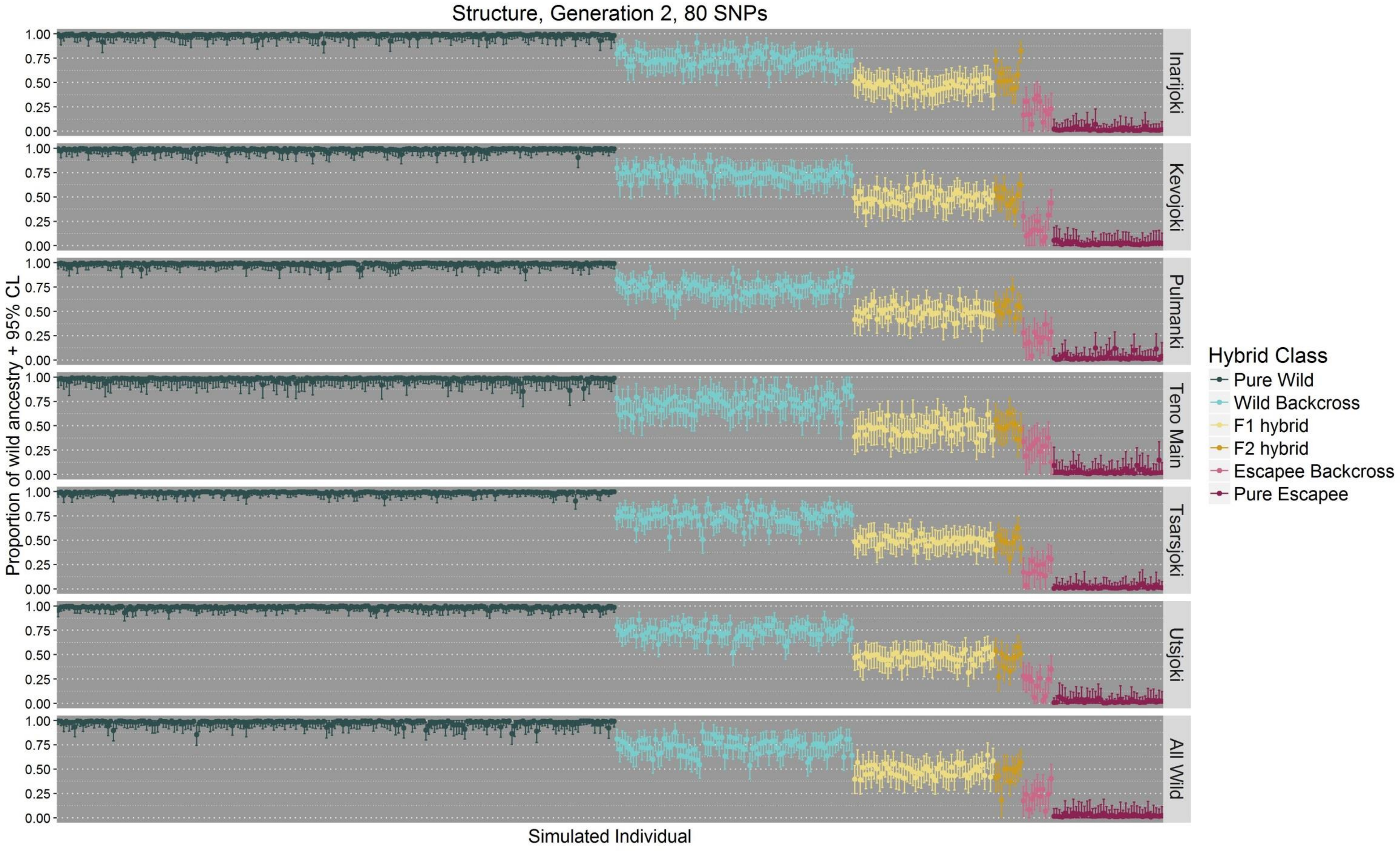

Structure, Generation 2, 80 SNPs
Proportion of wild ancestry + 95% CL
1.00
0.75
0.50
0.25
0.00
Inarijoki
Kevojoki
Pulmanki
Teno Main
Tsarsjoki
Utsjoki
All Wild
Simulated Individual
Hybrid Class
Pure Wild
Wild Backcross
F1 hybrid
F2 hybrid
Escapee Backcross
Pure Escapee

Figure S5e

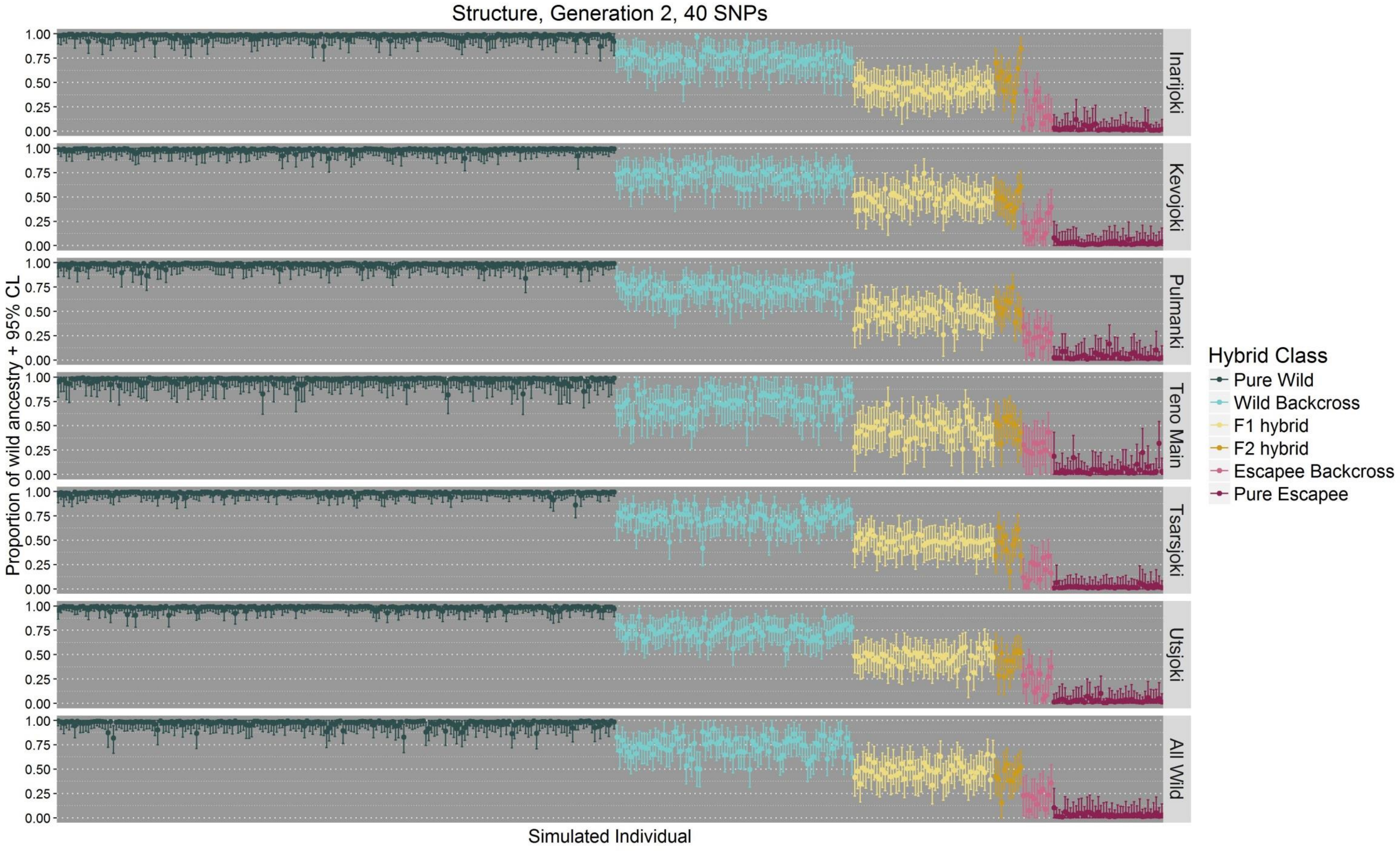

Structure, Generation 2, 40 SNPs
Proportion of wild ancestry + 95% CL
1.00
0.75
0.50
0.25
0.00
Inarijoki
Kevojoki
Pulmanki
Teno Main
Tsarsjoki
Utsjoki
All Wild
Simulated Individual
Hybrid Class
Pure Wild
Wild Backcross
F1 hybrid
F2 hybrid
Escapee Backcross
Pure Escapee

Figure S6a

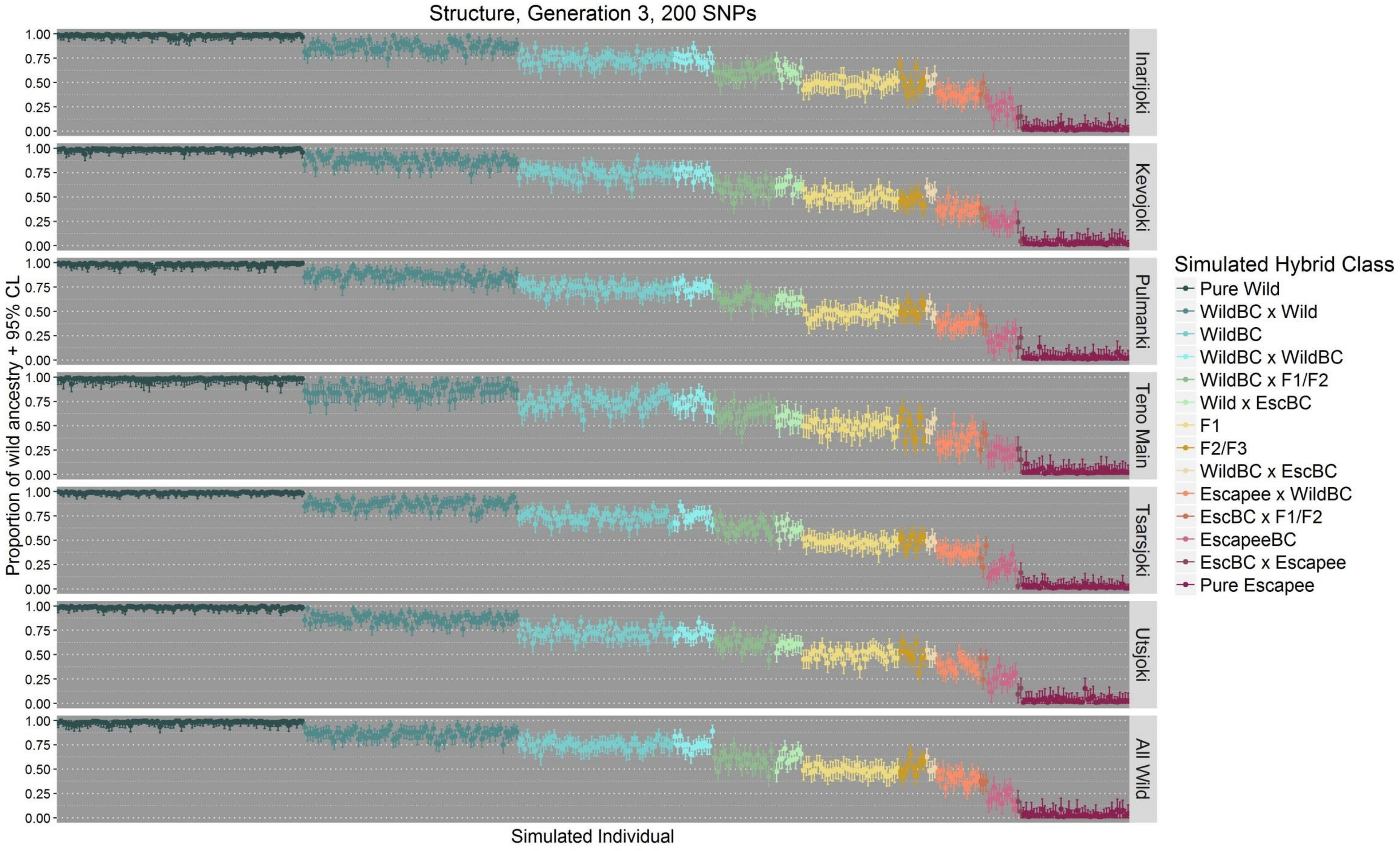

Structure, Generation 3, 200 SNPs
Proportion of wild ancestry + 95% CL
1.00
0.75
0.50
0.25
0.00
Inarijoki
Kevojoki
Pulmanki
Teno Main
Tsarsjoki
Utsjoki
All Wild
Simulated Individual
Simulated Hybrid Class
Pure Wild
WildBC x Wild
WildBC
WildBC x WildBC
WildBC x F1/F2
Wild x EscBC
F1
F2/F3
WildBC x EscBC
Escapee x WildBC
EscBC x F1/F2
EscapeeBC
EscBC x Escapee
Pure Escapee

Figure S6b

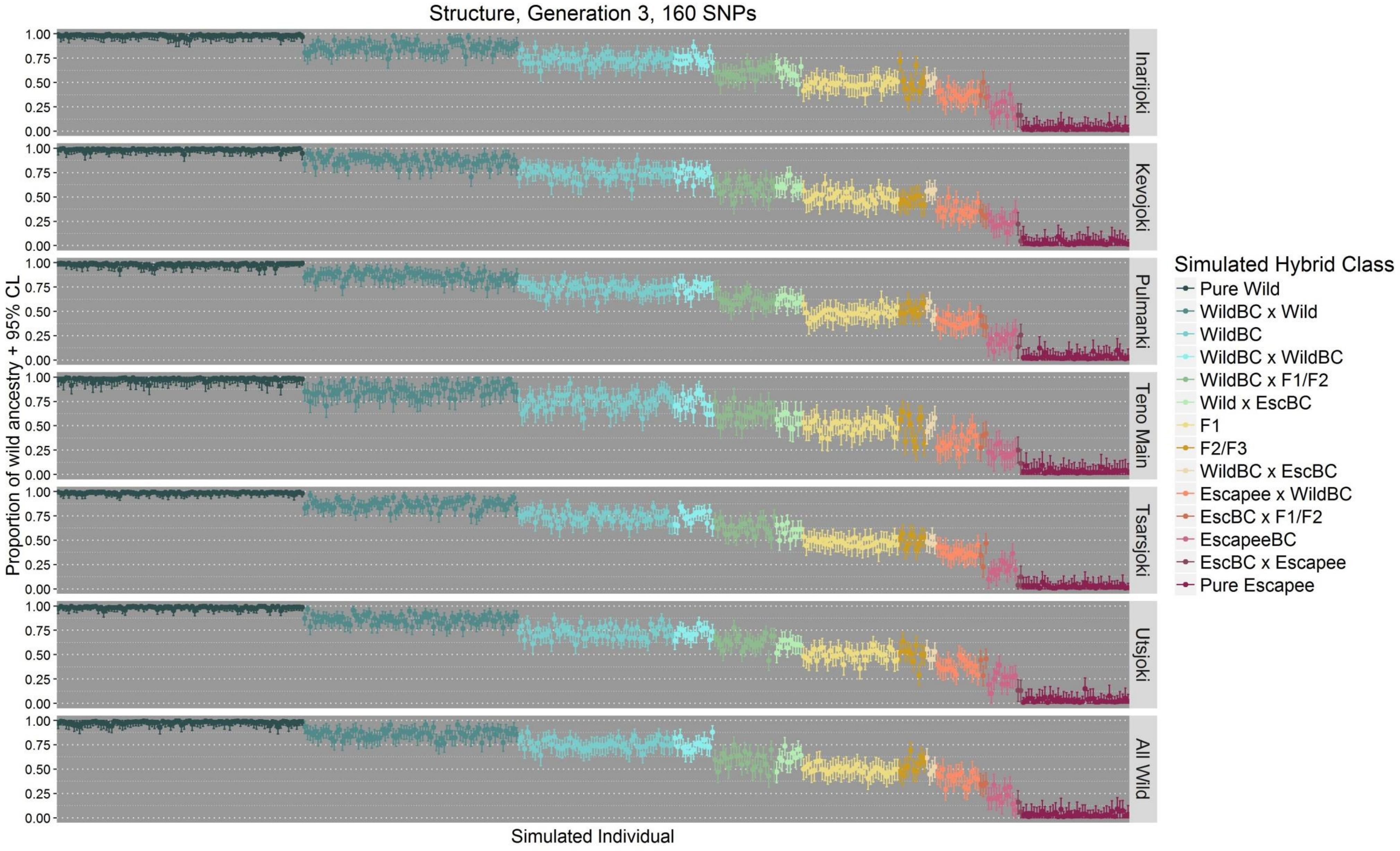
Structure, Generation 3, 160 SNPs
Proportion of wild ancestry + 95% CL
1.00
0.75
0.50
0.25
0.00
Inarijoki
Kevojoki
Pulmanki
Teno Main
Tsarsjoki
Utsjoki
All Wild
Simulated Individual
Simulated Hybrid Class
Pure Wild
WildBC x Wild
WildBC
WildBC x WildBC
WildBC x F1/F2
Wild x EscBC
F1
F2/F3
WildBC x EscBC
Escapee x WildBC
EscBC x F1/F2
EscapeeBC
EscBC x Escapee
Pure Escapee

Figure S6c

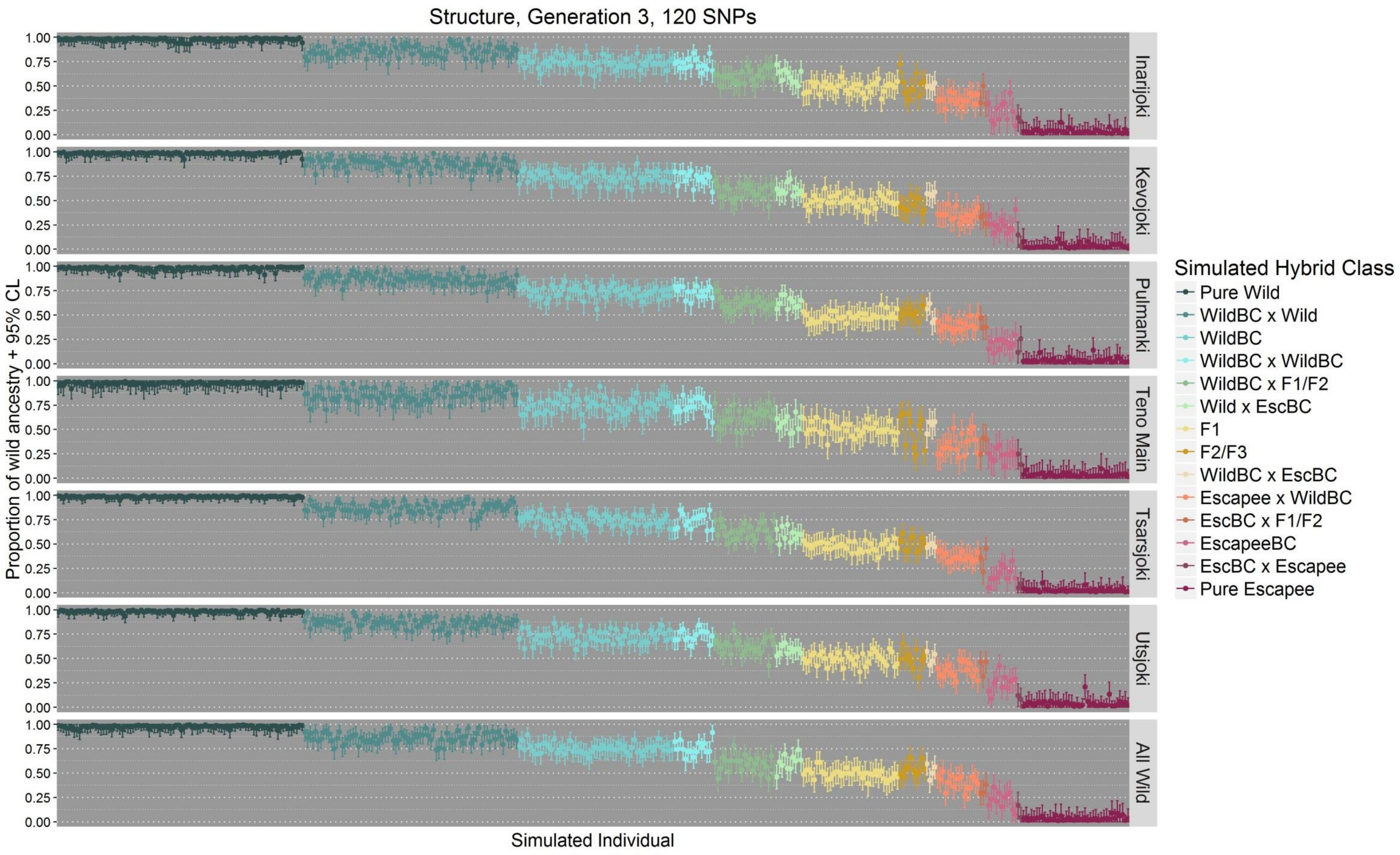

Structure, Generation 3, 120 SNPs
Proportion of wild ancestry + 95% CL
Simulated Individual
1.00
0.75
0.50
0.25
0.00
Inarijoki
Kevojoki
Pulmanki
Teno Main
Tsarsjoki
Utsjoki
All Wild
Simulated Hybrid Class
Pure Wild
WildBC x Wild
WildBC
WildBC x WildBC
WildBC x F1/F2
Wild x EscBC
F1
F2/F3
WildBC x EscBC
Escapee x WildBC
EscBC x F1/F2
EscapeeBC
EscBC x Escapee
Pure Escapee

Figure S6d

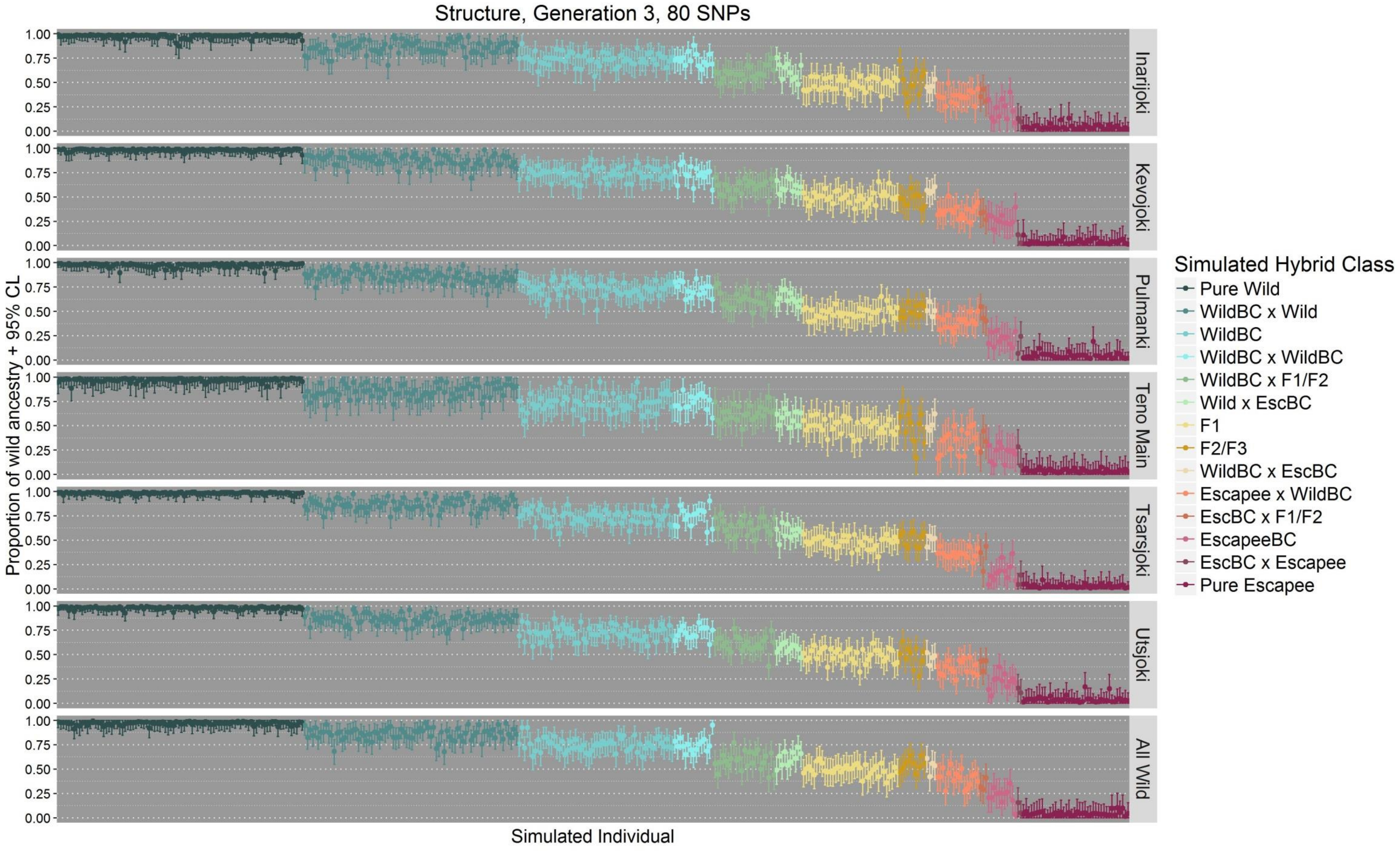
Structure, Generation 3, 80 SNPs
Proportion of wild ancestry + 95% CL
1.00
0.75
0.50
0.25
0.00
Simulated Individual
Inarijoki
Kevojoki
Pulmanki
Teno Main
Tsarsjoki
Utsjoki
All Wild
Simulated Hybrid Class
Pure Wild
WildBC x Wild
WildBC
WildBC x WildBC
WildBC x F1/F2
Wild x EscBC
F1
F2/F3
WildBC x EscBC
Escapee x WildBC
EscBC x F1/F2
EscapeeBC
EscBC x Escapee
Pure Escapee

Figure S6e

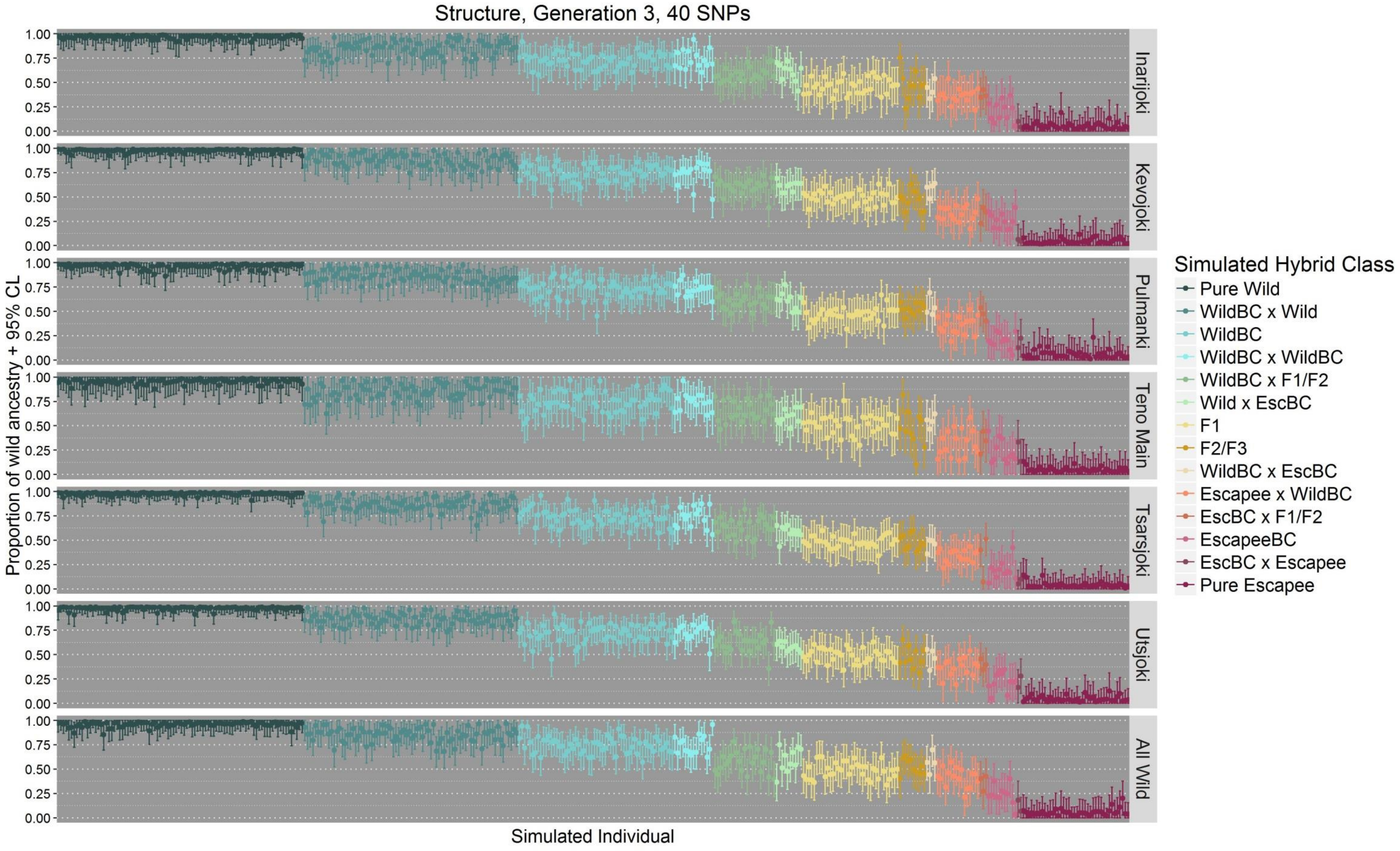

Structure, Generation 3, 40 SNPs
Proportion of wild ancestry + 95% CL
1.00
0.75
0.50
0.25
0.00
Inarijoki
Kevojoki
Pulmanki
Teno Main
Tsarsjoki
Utsjoki
All Wild
Simulated Individual
Simulated Hybrid Class
Pure Wild
WildBC x Wild
WildBC
WildBC x WildBC
WildBC x F1/F2
Wild x EscBC
F1
F2/F3
WildBC x EscBC
Escapee x WildBC
EscBC x F1/F2
EscapeeBC
EscBC x Escapee
Pure Escapee
