# Supplementary material for: Single nucleotide polymorphisms to discriminate different classes of hybrid between wild Atlantic salmon and aquaculture escapees
Source: Evol Appl. 2016 Aug 18;9(8):1017–31. doi: 10.1111/eva.12407 (PMC4999531; doi:10.1111/eva.12407)
Supplement: Supplementary file 5 [file EVA-9-1017-s005.pdf]

SNPs to discriminate different classes of hybrid between wild Atlantic salmon and aquaculture escapees: Supplementary Figures.

Figure S7 (a-d) Results of NewHybrids analyses for 400 individuals produced by two or three generations of simulated hybridization between aquaculture escapees and wild fish from Näätämö or Tornio. Different analyses were performed using different numbers of SNPS (40 – 200). For further details see previous figure legends.

Figure S8: Overall performance of different numbers of SNPs for correctly assigning different hybrid classes generated by hybridization between escapees and wild fish from Näätämö (top) or Tornio (bottom). Assignment was performed using NewHybrids.

Figure S7a

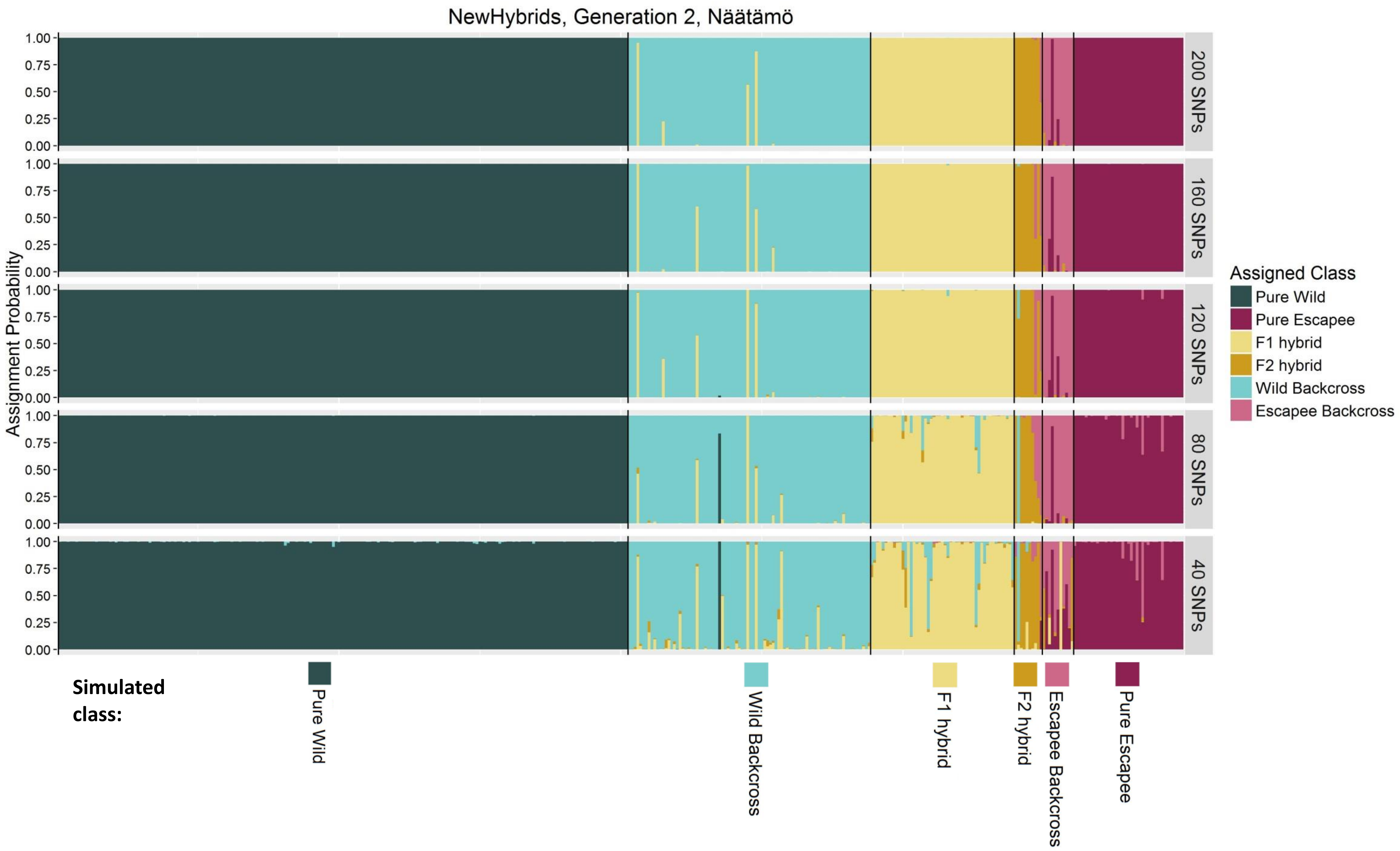

NewHybrids, Generation 2, Näätämö
Assignment Probability
1.00
0.75
0.50
0.25
0.00
200 SNPs
160 SNPs
120 SNPs
80 SNPs
40 SNPs
Assigned Class
Pure Wild
Pure Escapee
F1 hybrid
F2 hybrid
Wild Backcross
Escapee Backcross
Simulated class:
Pure Wild
Wild Backcross
F1 hybrid
F2 hybrid
Escapee Backcross
Pure Escapee

Figure S7b

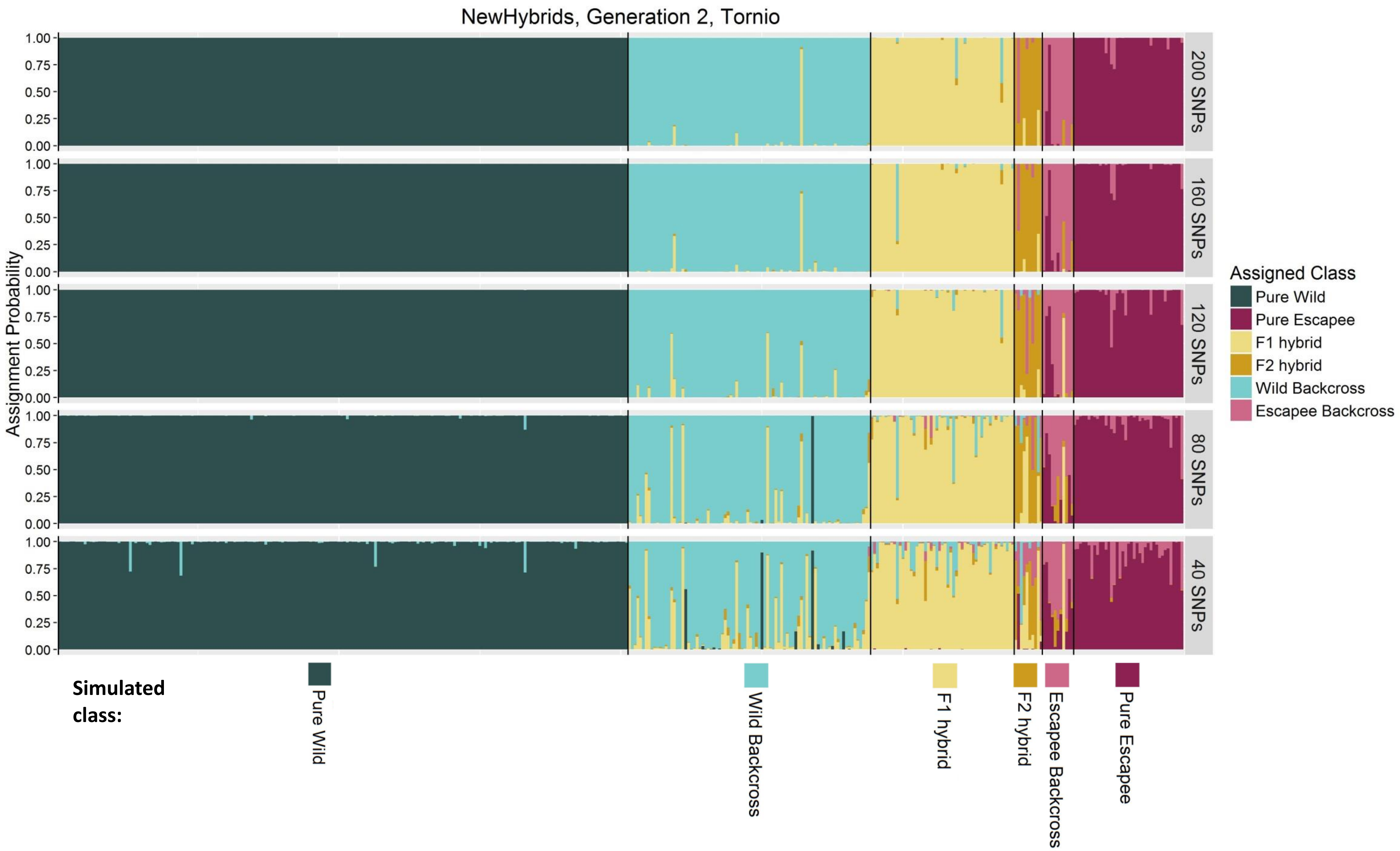

NewHybrids, Generation 2, Tornio
Assignment Probability
1.00
0.75
0.50
0.25
0.00
200 SNPs
160 SNPs
120 SNPs
80 SNPs
40 SNPs
Assigned Class
Pure Wild
Pure Escapee
F1 hybrid
F2 hybrid
Wild Backcross
Escapee Backcross
Simulated class:
Pure Wild
Wild Backcross
F1 hybrid
F2 hybrid
Escapee Backcross
Pure Escapee

Figure S7c

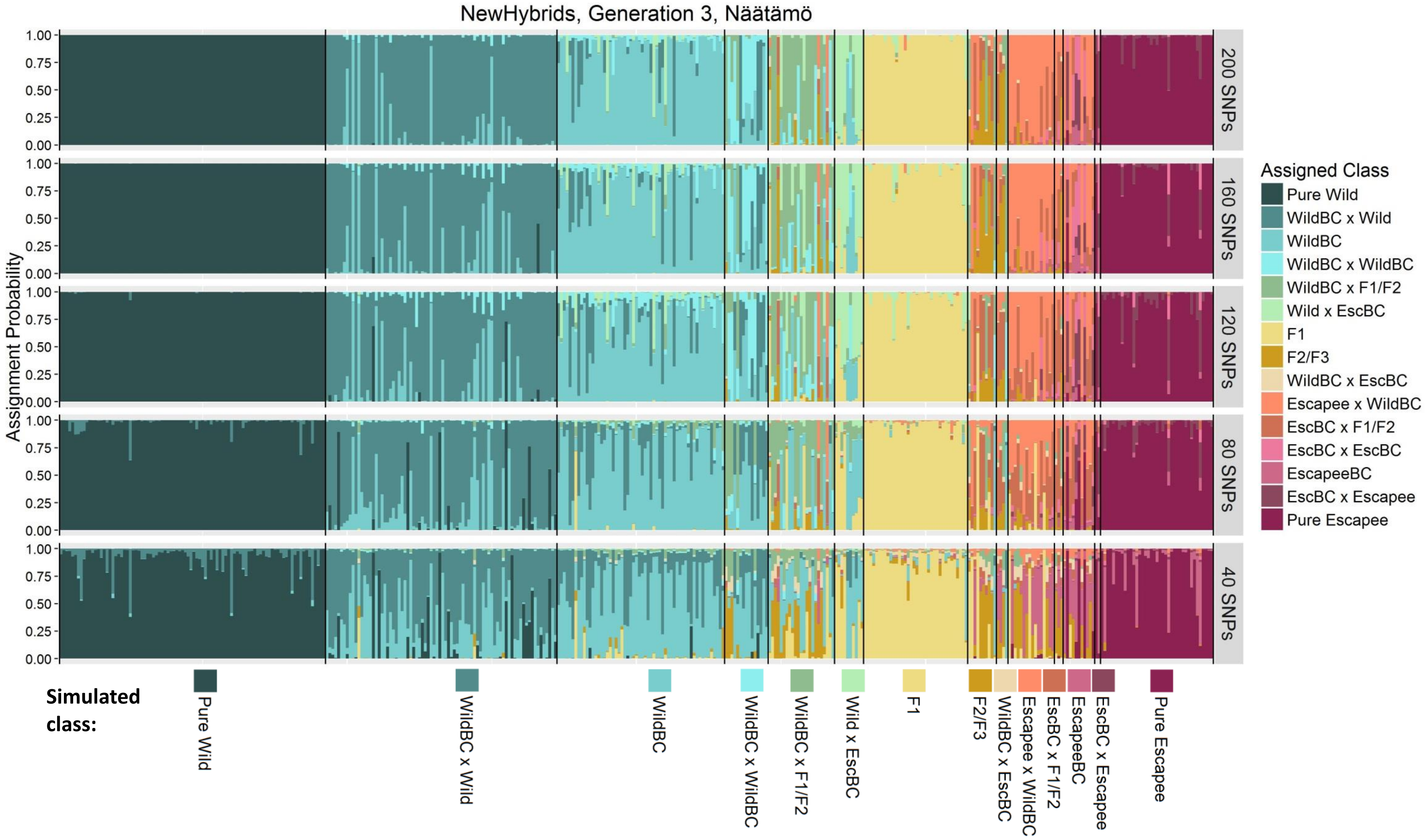
NewHybrids, Generation 3, Näätämö
Assignment Probability
1.00
0.75
0.50
0.25
0.00
200 SNPs
160 SNPs
120 SNPs
80 SNPs
40 SNPs
Assigned Class
Pure Wild
WildBC x Wild
WildBC
WildBC x WildBC
WildBC x F1/F2
Wild x EscBC
F1
F2/F3
WildBC x EscBC
Escapee x WildBC
EscBC x F1/F2
EscBC x EscBC
EscapeeBC
EscBC x Escapee
Pure Escapee
Simulated class:
Pure Wild
WildBC x Wild
WildBC
WildBC x WildBC
WildBC x F1/F2
Wild x EscBC
F1
F2/F3
WildBC x EscBC
Escapee x WildBC
EscBC x F1/F2
EscapeeBC
EscBC x Escapee
Pure Escapee

Figure S7c

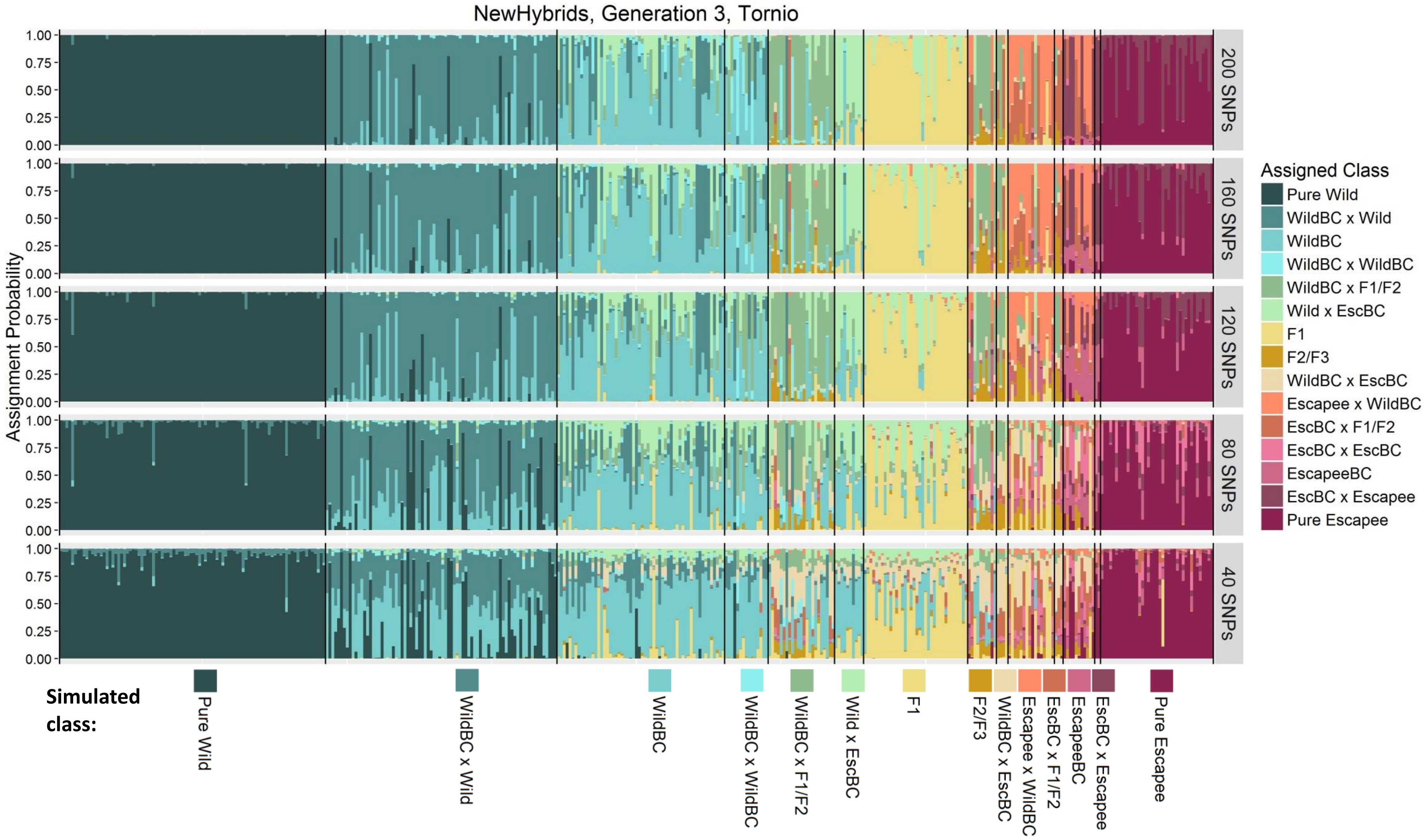
NewHybrids, Generation 3, Tornio
Assignment Probability
1.00
0.75
0.50
0.25
0.00
200 SNPs
160 SNPs
120 SNPs
80 SNPs
40 SNPs
Assigned Class
Pure Wild
WildBC x Wild
WildBC
WildBC x WildBC
WildBC x F1/F2
Wild x EscBC
F1
F2/F3
WildBC x EscBC
Escapee x WildBC
EscBC x F1/F2
EscBC x EscBC
EscapeeBC
EscBC x Escapee
Pure Escapee
Simulated class:
Pure Wild
WildBC x Wild
WildBC
WildBC x WildBC
WildBC x F1/F2
Wild x EscBC
F1
F2/F3
WildBC x EscBC
Escapee x WildBC
EscBC x F1/F2
EscapeeBC
EscBC x Escapee
Pure Escapee

Figure S8

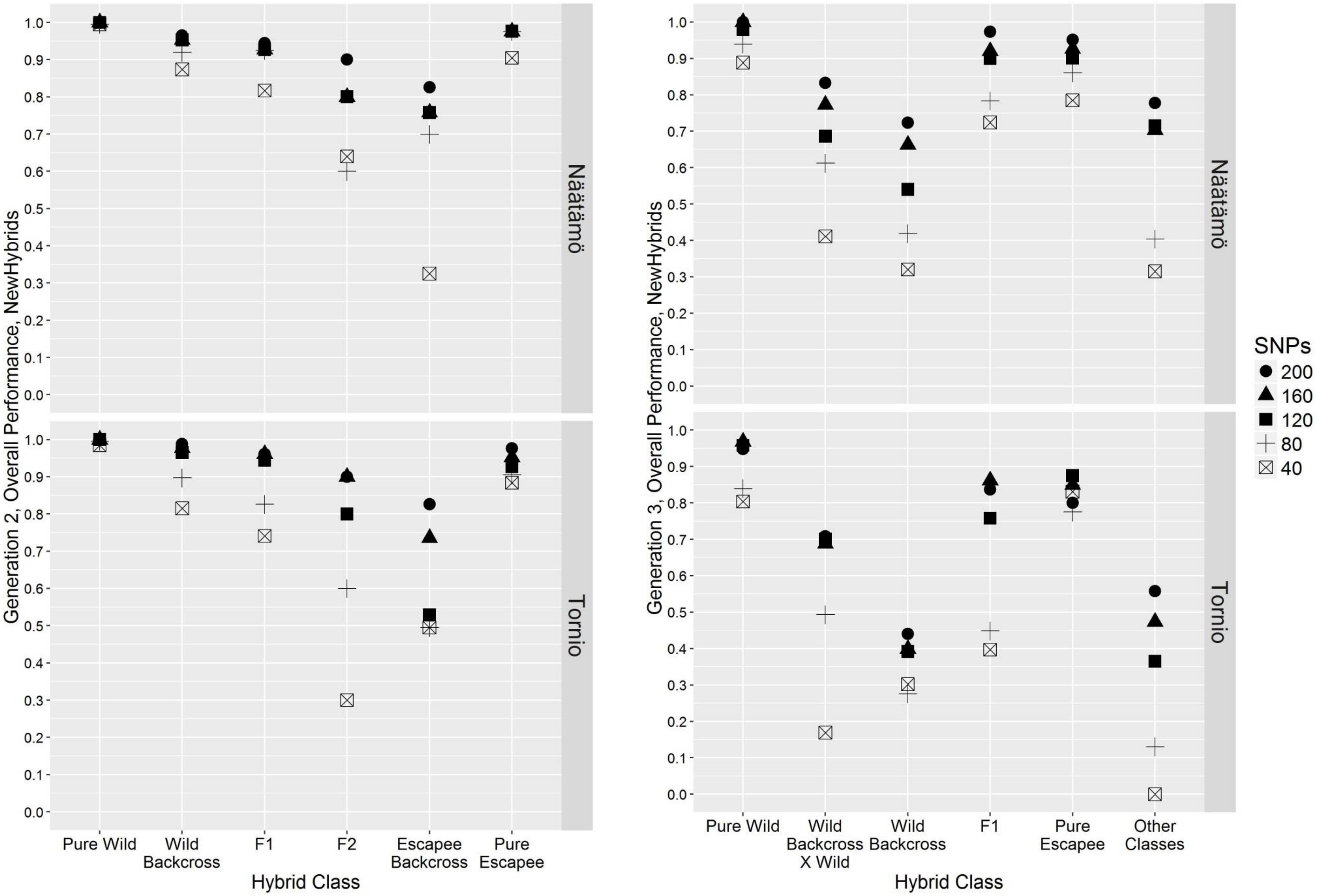
Generation 2, Overall Performance, NewHybrids
Näätämö
Tornio
Pure Wild
Wild Backcross
F1
F2
Escapee Backcross
Pure Escapee
Hybrid Class
Generation 3, Overall Performance, NewHybrids
Wild Backcross X Wild
Wild Backcross
Other Classes
SNPs
200
160
120
80
40
