# Supplementary material for: Single nucleotide polymorphisms to discriminate different classes of hybrid between wild Atlantic salmon and aquaculture escapees
Source: Evol Appl. 2016 Aug 18;9(8):1017–31. doi: 10.1111/eva.12407 (PMC4999531; doi:10.1111/eva.12407)

SNPs to discriminate different classes of hybrid between wild Atlantic salmon and aquaculture escapees: Supplementary Figures.

Figure S9: Multidimensional scaling analysis plot visualizing genome-wide identity by state amongst Old Teno Mainstem, New Teno Mainstem, Teno Escapee, and Tornio samples. Analysis is based on 49,086 SNPs remaining after excluding SNPs with >2% missing data and performing LD-based pruning. Each point represents an individually genotyped fish. Arrows indicate three possibly mis-identified 'aquaculture escapees' removed from subsequent analyses.

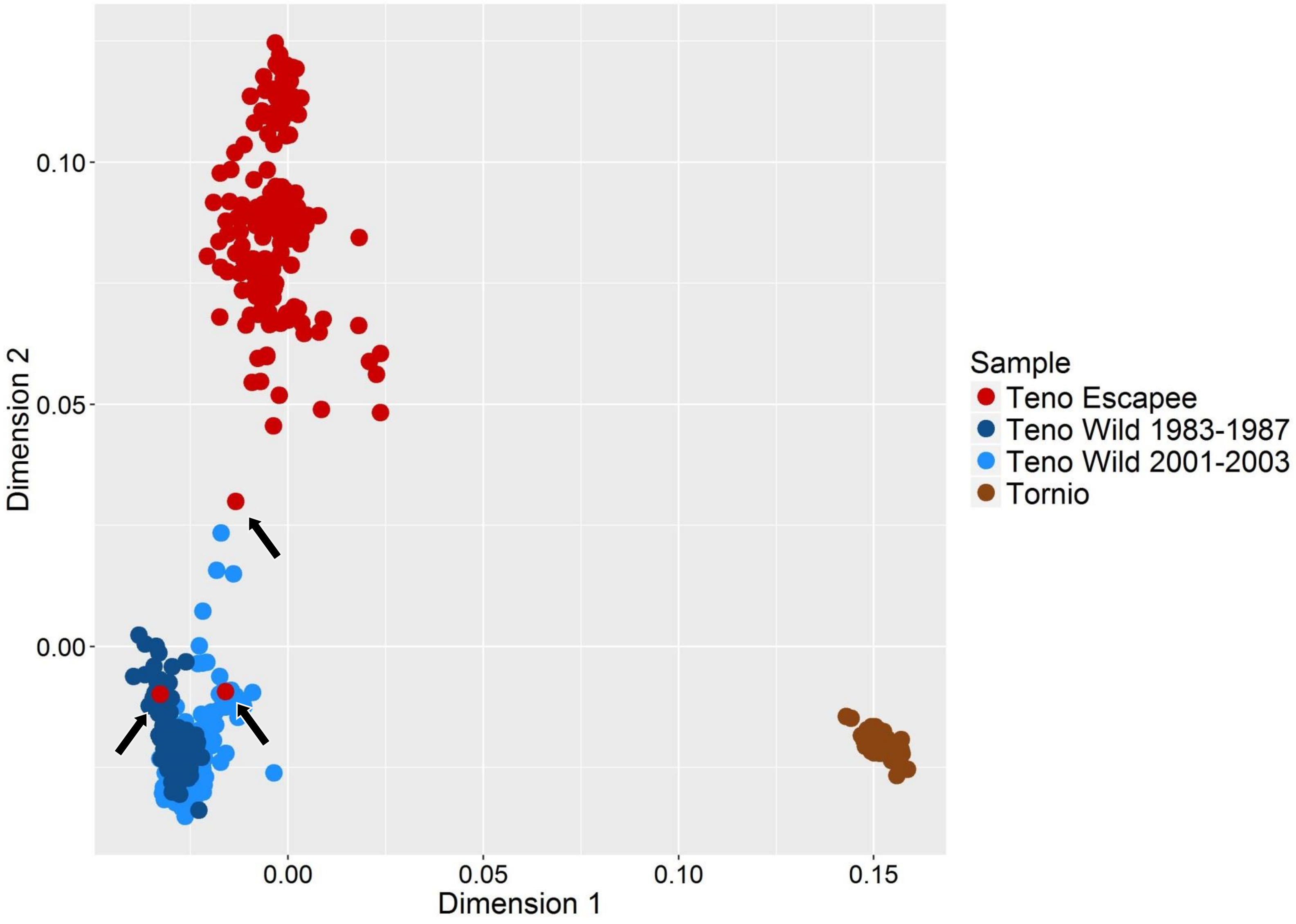

Supplement: Supplementary file 6 [file EVA-9-1017-s006.pdf]
